# Potential Novel Biomarkers in Chronic Graft-Versus-Host Disease

**DOI:** 10.3389/fimmu.2020.602547

**Published:** 2020-12-23

**Authors:** Rachel E. Crossland, Francesca Perutelli, Katarzyna Bogunia-Kubik, Nuala Mooney, Nina Milutin Gašperov, Maja Pučić-Baković, Hildegard Greinix, Daniela Weber, Ernst Holler, Dražen Pulanić, Daniel Wolff, Anne M. Dickinson, Marit Inngjerdingen, Magdalena Grce

**Affiliations:** ^1^Translational and Clinical Research Institute, Faculty of Medical Sciences, Newcastle University, Newcastle upon Tyne, United Kingdom; ^2^Department of Molecular Biotechnology and Health Sciences, School of Medicine, University of Torino, Torino, Italy; ^3^Department of Clinical Immunology, Hirszfeld Institute of Immunology and Experimental Therapy, Polish Academy of Sciences, Wroclaw, Poland; ^4^INSERM U976, Human Immunology, Pathophysiology and Immunotherapies, Hôpital Saint Louis, Paris, France; ^5^Division of Molecular Medicine, Ruđer Bošković Institute, Zagreb, Croatia; ^6^Genos, Glycoscience Research Laboratory, Zagreb, Croatia; ^7^Division of Hematology, Department of Internal Medicine, Medical University of Graz, Graz, Austria; ^8^Department of Internal Medicine III, Faculty of Medicine, University Hospital Regensburg, Regensburg, Germany; ^9^Division of Hematology, Department of Internal Medicine, University Hospital Centre Zagreb, Medical School, University of Zagreb, Zagreb, Croatia; ^10^Department of Pharmacology, Institute of Clinical Medicine, University of Oslo and Oslo University Hospital, Oslo, Norway

**Keywords:** chronic graft-versus-host disease (cGvHD), alloantibodies, glycomics, endothelial derived particles, extracellular vesicles, epigenetic changes, microbiome, cellular biomarkers

## Abstract

Prognostic, diagnostic or predictive biomarkers are urgently needed for assessment of chronic graft-versus-host disease (cGvHD), a major risk for patients undergoing allogeneic hematopoietic stem cell transplantation. The main goal of this review generated within the COST Action EUROGRAFT “Integrated European Network on Chronic Graft Versus Host Disease” was to identify potential novel biomarkers for cGvHD besides the widely accepted molecular and cellular biomarkers. Thus, the focus was on cellular biomarkers, alloantibodies, glycomics, endothelial derived particles, extracellular vesicles, microbiome, epigenetic and neurologic changes in cGvHD patients. Both host-reactive antibodies in general, and particularly alloantibodies have been associated with cGvHD and require further consideration. Glycans attached to IgG modulate its activity and represent a promising predictive and/or stratification biomarker for cGVHD. Furthermore, epigenetic changes such as microRNAs and DNA methylation represent potential biomarkers for monitoring cGvHD patients and novel targets for developing new treatment approaches. Finally, the microbiome likely affects the pathophysiology of cGvHD; bacterial strains as well as microbial metabolites could display potential biomarkers for dysbiosis and risk for the development of cGvHD. In summary, although there are no validated biomarkers currently available for clinical use to better inform on the diagnosis, prognosis or prediction of outcome for cGvHD, many novel sources of potential markers have shown promise and warrant further investigation using well characterized, multi-center patient cohorts.

## Introduction

**Figure 1:**
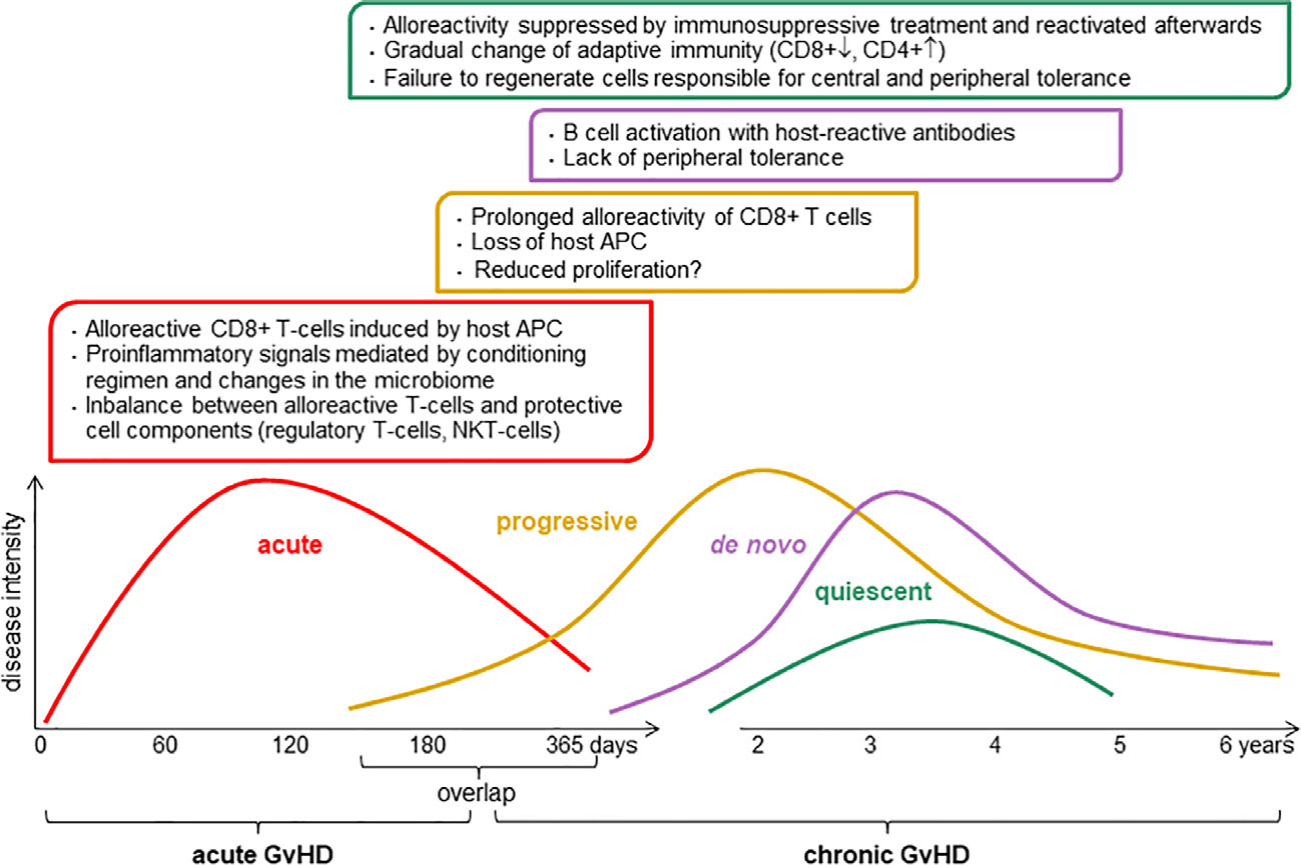
Pathophysiology of chronic graft-versus-host disease (GvHD). The intensity and the length of GvHD is multifactorial, it depends of conditioning regimen, donor and host status including graft source, donor type, HLA match, age, and gender. APC, antigen-presenting cell; CD8^+^, cytotoxic T cells; CD4^+^, helper T cells; NKT, natural killer T cells.

Chronic graft-versus-host disease (cGvHD) is a major risk for patients undergoing allogeneic hematopoietic stem cell transplantation (alloHSCT). It is a multi-organ autoimmune disorder and is the major cause of non-relapse morbidity and mortality after alloHSCT, occurring in about 50% of patients, or 13,000–15,000 patients per year worldwide (1). GvHD develops when mature immunocompetent donor T cells present in the graft recognize alloantigenes expressed by the recipient (2). Several factors influence the course of immunoreconstitution which either can lead to: 1) normal immune restoration of protective immunity with host tolerance, 2) functional tolerance with graft-versus-tumor effects, or 3) immune dysregulation and alloreactivity that consequently causes aGvHD and/or later chronic GvHD (**Figure 1**). Therefore, there is a urgent medical need for immune dysregulation leading to manifestation of GvHD (1).

The diagnosis of cGvHD, according to the National Institutes of Health (NIH) consensus criteria, can be made if there is presence of a diagnostic feature, or if there is at least one distinctive manifestation in addition to radiologic, histologic or laboratory evidence of GvHD from any site (3). Transplant recipients with cGvHD have a reduced quality of life and increased risks of long-term morbidity and mortality, in comparison with transplant recipients who do not develop cGvHD. Chronic GvHD can involve not only the epithelial target tissues affected in classic acute GvHD (aGvHD); gastrointestinal tract, liver, skin, and lungs, but also any other organ system, including oral, esophageal, musculoskeletal, joint, fascial, ocular, genital, peripheral nervous and lymphohematopoietic systems. Organ involvement is more heterogeneous and disease manifestations are more variable in cGvHD compared to aGvHD.

Although some novel therapeutic approaches have shown a good efficacy in cGvHD therapy, it is unlikely that they will completely overcome drug resistance, so combined therapies could be promising in the next generation of trials. The value of potential cGvHD biomarkers is in their usefulness for prognosis, predicting therapeutic responses, and for identifying new therapeutic targets (1). Identification of cGvHD biomarkers requires speciﬁc consideration of the sensitivity and speciﬁcity in subgroups with different clinical characteristics. Any biomarker should be carefully evaluated during the veriﬁcation phase. In summary, a joint effort is required to verify the results of numerous trials before any of the potential candidate biomarkers can progress to validation and clinical application (4).

Biomarkers for cGvHD may be classified as prognostic, diagnostic or predictive. Each has their own importance and clinical relevance, while also presenting challenges to researchers related to their identification and validation. A prognostic biomarker provides information about the patients’ overall outcome, regardless of therapy, and thus may propose the further course of disease or onset of subsequent GvHD. In order for prognostic markers to be clinically relevant, high negative prediction with regard to the distinction between moderate/severe *versus* mild or no GvHD is of great importance. Despite this challenge, prognostic cGvHD biomarkers may be considered as the area of greatest medical need, particularly as a guide to taper immunosuppression. Diagnostic biomarkers can be used to confirm the presence of cGvHD and/or to differentiate from active aGvHD or other conditions. These biomarkers may accelerate diagnosis in the clinic, potentially by adding to or replacing histopathological approaches. This may be of particular advantage in clinical trials and pediatric patients. Predictive biomarkers are necessary to predict response or lack of response to treatment, including mortality. However, all biomarkers are associated with several limitations and caveats. To date, no cGvHD biomarkers have been reliably replicated in independent studies and as a consequence, there are no approved clinical grade tests. This may be fuelled by insufficient characterization of cGvHD clinical subtypes, as well as ambiguity in relation to aGvHD overlap and biological definitions of such overlap. The small sample size and heterogeneity of assays in clinical trials as well as heterogeneous time points analyzed has further resulted in inconsistent capture or reporting of biomarkers and associated cofactors.

In this review paper, we performed a literature review using the search terms “chronic graft-versus-host disease,” “cellular biomarkers,” “alloantibodies,” “glycomics,” “endothelial derived particles,” “extracellular vesicles,” “microRNA,” “DNA methylation,” and “microbiome.” We excluded the genetic variabilities associated with cGvHD, which are extensively reviewed in another review paper (5) of the collection of publications generated by the COST Action EUROGRAFT “Integrated European Network on Chronic Graft Versus Host Disease” (CA17138). Thus, in this paper we focused on potential future novel, still unexplored biomarkers, which could help to better understand and manage cGvHD patients such as cellular biomarkers, alloantibodies, endothelial derived particles and microbiome as well as the epigenetic changes in cGvHD patients.

## Cellular Biomarkers for cGvHD

Phenotypic patterns of cGvHD (**Figure 1**) may be classified into inflammatory and sclerotic presentations with immune dysfunction as hallmark of cGvHD (6). Preclinical studies and translational research on human biospecimens have implicated certain biological pathways in the pathophysiology of cGvHD, leading to exploration of immune cell-derived diagnostic, prognostic and predictive biomarkers in both hypothesis-driven and discovery-based testing (6, 7).

Donor B cells contribute substantially to the development of cGvHD and both B cell activating factor (BAFF) and B cell receptor (BCR) signaling play critical roles in this process. BAFF promotes survival and differentiation of allo- and autoreactive B cells. Sarantopoulos and colleagues observed an altered B cell homeostasis and excess of BAFF in patients developing cGvHD (8). Furthermore, an increased BCR responsiveness that could be abrogated by Syk inhibition was reported in B cells of patients with cGvHD (9, 10). Of note, inhibition of Syk by fostamatinib decreased cGvHD pathology in a murine bronchiolitis obliterans syndrome (BOS) model and induced apoptosis in B cells of patients with cGvHD, demonstrating that B cell activation is of importance for development of cGvHD (11). The CD19^+^CD21^−^ subpopulation of B cells has been reported as potential prognostic and diagnostic cellular biomarker of cGvHD correlating with disease severity and organ manifestations (12–14). Assessment of B cell subpopulations allowed a distinction of different impairments of humoral immunity seen as either immunodeficiency or autoimmunity (13). In a prospective study including 136 patients (46 BOS, 41 no cGvHD, 49 cutaneous cGvHD) to define novel biomarkers for early diagnosis of NIH-defined BOS, diagnosed a median of 11 months after HSCT patients with newly diagnosed BOS had significantly higher percentages of CD19^+^CD21^low^ B cells, BAFF levels, and BAFF/CD19^+^ ratios compared with patients without cGvHD. This supports CD19^+^CD21^low^ B cells as a potential novel biomarker for HSCT patients at risk for developing BOS (14).

In serial analyses starting on day +100 after HSCT and including 163 patients with cGvHD and 64 never experiencing cGvHD and sampled as time-matched controls, Greinix and colleagues reported that elevated frequencies of CD19^+^CD21^-^ B cells significantly correlated with first diagnosis of cGvHD (15). Furthermore, elevation of CD19^+^CD21^-^ B cells on day +100 after HSCT was associated with later development of cGvHD and could serve as a prognostic biomarker for clinically significant cGvHD and for quiescent and progressive onset type of cGvHD, respectively. Circulating toll-like receptor 9 expressing B cells have also been associated with the development of cGvHD in patients after transplant (16).

Regulatory B (Breg) cells are immunosuppressive cells that support immunological tolerance. Different B cell subpopulations are reportedly enriched in Breg cells including CD24^hi^CD27^+^, CD24^hi^CD38^hi^ transitional B cells, and CD20^+^CD27^+^CD43^+^ CD70^+^ B cells and different inflammatory environments could induce distinct Breg cell populations (17). Khoder and colleagues observed an enrichment of Breg cells in patients with cGvHD within both CD19^+^IgM^+^CD27^+^ memory and CD19^+^CD24^hi^ CD38^hi^ transitional B cell subsets (18). Furthermore, Breg cells from patients with cGvHD were less frequent and less likely to produce interleukin-10. De Masson and colleagues reported a decreased Breg cell frequency in patients with active cGvHD and a correlation of Breg cells with severity of cGvHD (19).

Regulatory T (Treg) cells that are essential for establishment and maintenance of tolerance after HSCT reportedly were significantly reduced in patients with cGvHD (20–22). Detrimental factors for Treg cells in cGvHD have been recognized in diminished thymic production, reduced proliferative capacity of Treg cells and increased susceptibility to apoptosis of memory Treg cells (21, 22). Donor T effector cells have been shown to mediate alloreactivity toward recipient tissue in both human and mice (6) and are important for cGvHD development. Studies have demonstrated a role for Th17 cells in murine models of autoimmunity, scleroderma, and multiorgan system disease with BOS (23, 24). Dander and colleagues observed an increase of Th17 cells in the blood of patients with active cGvHD (25). In the skin of patients with lichenoid cGvHD, a Th1/Th17 signature has been reported (26), whereas in the oral mucosa of patients with cGvHD increased T-bet+ cytotoxic effector cells and type I interferon-mediated processes have been observed (27). Forcade and colleagues reported an increased frequency of CD4^+^CD146^+^CCR5^+^ T cells, a Th17 prone subpopulation of CD4^+^ T cells, in patients with active cGvHD (28). In a murine cGvHD model with BOS, donor T cells from CD146-deficient mice caused significantly less pulmonary cGvHD with lower pulmonary macrophage infiltration and T cell CCR5, IL-17, and interferon-γ coexpression. Lower circulating T follicular helper (TFH) cells have also been observed in patients with active cGvHD compared to patients without cGvHD (29). Furthermore, the phenotype of CD4^+^TFH cells (CD4^+^CD45RA^−^CXCR5^+^) was skewed toward a highly activated profile with predominance of Th2/Th17 subsets, while activated CD4^+^TFH in cGvHD patients had an increased functional ability to promote B cell immunoglobulin secretion and maturation.

Lack of CXCR3^+^ CD56^bright^ natural killer (NK) cells correlated with diagnosis of cGvHD (30). Immune profiling using peripheral blood samples of 302 children including late-aGvHD, cGvHD and no cGvHD revealed increased cytolytic NK cells, increased activated T cells, naive helper T and cytotoxic T cells, loss of CD56^bright^ regulatory NK cells, and increased ST2 and soluble CD13 (31). CD3^+^CD56^+^ NKT cells reportedly were also elevated in patients on day +100 after HSCT who later developed cGvHD (15).

In summary, very few cellular biomarkers have been identified from clinical studies, including discovery and independent verification as requested by the NIH consensus development group (4, 7). It will be challenging to verify and qualify the candidate biomarkers identified in previous studies and close cooperation of clinical and laboratory-based research groups will be necessary to pursue such studies successfully.

## Alloantibodies and GvHD

Donor-specific antibodies (DSA) have been strongly linked with chronic humoral rejection in solid organ transplantation and the robust association between complement activating DSA and graft failure has been previously demonstrated (32). DSA mediated pathways of allograft damage, that are independent of complement activation, have also been revealed (33–35). However, as many of the studies investigating alloantibodies, and particularly DSA, have been carried out in renal transplantation, much of our current understanding of their role stems from these studies. The effect of DSA has not been evaluated to the same degree following HSCT, compared to in organ transplantation. This was initially because of the high degree of HLA matching between donor and recipient in HSCT.

Similarly to the situation in organ transplantation, anti-HLA antibodies may arise as a result of blood transfusions or pregnancies and in haploidentical HSCT, the prevalence of DSA was reported to be considerably higher in female recipients (36). Furthermore, DSA are an obstacle to engraftment, although this is not necessarily the case for anti-HLA antibodies overall (37). In addition, Ciurea et al. (38) reported that compared to male recipients, multiparous females had a higher prevalence of anti-HLA antibodies. Moreover, an increased risk of cGvHD after HLA-identical sibling HSCT was found after transplantation with female donors who had experienced one or more pregnancies (39). This observation was most probably due to previous female donor allosensitization.

The so-called natural anti-HLA antibodies can occur in low titers even in healthy people (e.g. in non-transfused males) or in cord blood (40, 41). Due to alloimmunization or earlier transfusions, their presence is more often found in patients with hematopoietic diseases. Anti-HLA antibodies occur in 12% to 42% of potential recipients and they are more often determined in women compared to men (30–42% *vs* 12%). Their levels are higher in women after one pregnancy, increases after repeated pregnancies, and mother’s antibodies are directed against HLA antigens in offspring (42, 43). In the case of alloHSCT, recipient B lymphocytes will stop producing anti-HLA against donor antigens only if complete chimerism is reached (*i.e.* the recipient’s hematopoietic system is fully restored with donor cells).

In a mouse model representing characteristic features of cGvHD such as multi-organ involvement, among the indications of cGvHD, fibrosis was observed in some organs and was associated with alloantibody deposition as well as CD4^+^ T and B cell infiltration. Because the prevention of germinal center formation suppressed GvHD and lung manifestations of disease, the authors concluded that cGvHD is at least partially due to alloantibody secretion (44).

Initial studies examined HLA-specific antibodies in pre-transplantation sera from patients undergoing bone marrow and cord blood HSCT. Relationships between the presence of anti-HLA antibodies and HSCT outcome were observed for patients transplanted with an unrelated donor (42, 45) and their results showed not only associations with DSA levels, but also with the presence of anti-HLA-DPB1 antibodies. Indeed, Spellman et al. (45) observed DSA in pretransplant sera of 24% of 37 patients, whereby DSA were mainly directed against HLA class I and HLA-DP antigens.

These results are not surprising, as the HLA-DP molecules are expressed on hematopoietic cells and, moreover, about 80% of donor-recipient pairs of unrelated HSCT are mismatched for at least 1 HLA-DPB1 antigen/allele. The latter is due to recombination hotspots that are located between HLA-DQB1 and HLA-DPB1 genes, and therefore matching patients and unrelated donors with common HLA-A, -C, -B, -DRB1, -DQB1 haplotypes (10/10 match) does not necessarily implicate matching for HLA-DPB1 and HLA-DPA1 alleles (reviewed in (46). Indeed, in unrelated donor-recipient settings, the presence of anti-HLA-DPB1 antibodies was clinically relevant with respect to development of transplant-related complications, including cGvHD. The outcome of having HLA antibodies in voluntary unrelated donors was analyzed by Delbos et al. (47). They observed a significantly higher cumulative incidence of cGvHD in recipients transplanted with anti-HLA class II immunized donors, directed mainly against HLA-DPB1 alleles. Moreover, they found that donor-derived anti-HLA antibodies could be detected up to 6 months after transplantation. Pan et al. (48) analyzed the presence of anti-HLA antibodies and monitored their dynamic changes after transplantation from unrelated donors. The presence of anti-HLA antibodies was found to be associated with increased risk of cGvHD, as well as higher aGvHD grades, and increased treatment-related mortality, while overall and disease-free survival were reduced. These data suggest that donor immunization against foreign HLA antigens may constitute a predictive marker of cGvHD occurrence after transplantation from HLA-mismatched unrelated donors.

Takanashi et al. (49) tested for DSA in pre-transplant sera from a series of 386 cord blood transplants and reported that DSA were associated with delayed neutrophil recovery post-transplantation. In a more recent study of a cohort of patients (n = 89) who had haploidentical donors with post-transplantation cyclophosphamide, 32 patients had anti-HLA antibodies, 12 with DSA and 8 of the 12 with DSA had complement activating antibodies (50).

Donor-Specific-Antibodies directed against HLA class I and class II antigens have been reported and in one large study of matched unrelated donor HSCT recipients (n=592), detection of DSA was the only factor that predicted graft failure in multivariate analysis (42). Another study (n=79) reported 11 patients with DSA and poorer neutrophil engraftment was associated with the presence of DSA (51). It is worth noting that the higher the level of circulating DSA detected, the poorer the engraftment. This leads to the suggestion that similarly to the situation in organ transplantation, DSA may, to some degree, be considered as biomarkers (albeit imperfect biomarkers). Refinement not only of the detection of DSA, but also of their characterization including their complement activating ability and/or ability to activate signaling within target cells may enhance their potential role as biomarkers in the HSCT setting. In addition, anti-HLA II antibodies have the ability to activate a cell death pathway and this should also be taken into consideration (52–54). A recent report extends this observation to DSA from patient allosera, revealing death of endothelial cells after DSA binding (55).

Previous studies of data from HSCT are contradictory with studies from renal transplantation, which has provided the most information to date about the prevalence and clinical implications of DSA. This is partly due to the lack of donor-matching for what have been described as low-expression loci; HLA-DP and -DQ. It is interesting to note that although HLA-DQ can be considered as a low-expression locus on the allograft endothelium in solid organ transplantation, it is the most frequently detected *de novo* DSA post-transplantation (reviewed in (56) and potential mechanisms of HLA-DQ alloantibody mediated damage to the allograft endothelium have been revealed (57). Moreover, the importance of pre-transplantation DSA anti-DQ has been recently reported to be independently associated with graft failure, and detection of DSA specific for HLA-DQ is a major predictor for graft failure (58). Analysis of DSA in HSCT is additionally complicated by the different sources of stem cells used in previous studies of HSCT, as well as different extents of HLA-loci matching.

There are studies showing the impact of genotyping and matching for non-classical HLA (including HLA-E, HLA-G or MICA/B loci) on HSCT outcome, including manifestation of cGvHD (reviewed in (46, 59, 60), and the role of anti-MICA antibodies in organ transplant recipients. Therefore, non-classical HLA molecules should also be considered as potential targets of alloantibodies in HSCT recipients. It has also been suggested that antibodies directed against minor histocompatibility antigens (mHA) may play a role in the development of cGvHD and other transplant-related complications. This effect was observed after transplantation from a haploidentical mother to her son, where anti-parental-mHA alloantibodies were found to persist for a long time post-transplantation (61).

Recent reports suggest that it may be insufficient to take into account only DSA directed against histocompatibility antigens, but that alloantibodies should be further characterized e.g. complement-binding DSA (C1q) may affect transplant outcome, as shown for haploidentical HSCT (38). In addition, in the setting of renal transplantation, recent data has revealed the importance of DSA directed against non-HLA antigens (62, 63). The contribution of DSA directed against non-histocompatibility antigens remains to be explored following HSCT.

In summary, at this stage, it is clear that a high level of DSA is a risk factor for HSCT transplantation and further characterization and identification of DSA would be helpful. While in the HLA-DP mismatched setting HLA directed alloantibodies may play a role in cGvHD, and this also applies to other HLA-mismatches, much of the antibody mediated damage in cGvHD derives from host reactive antibodies that are partly auto-reactive.

## Glycomics in cGvHD

**Figure 2:**
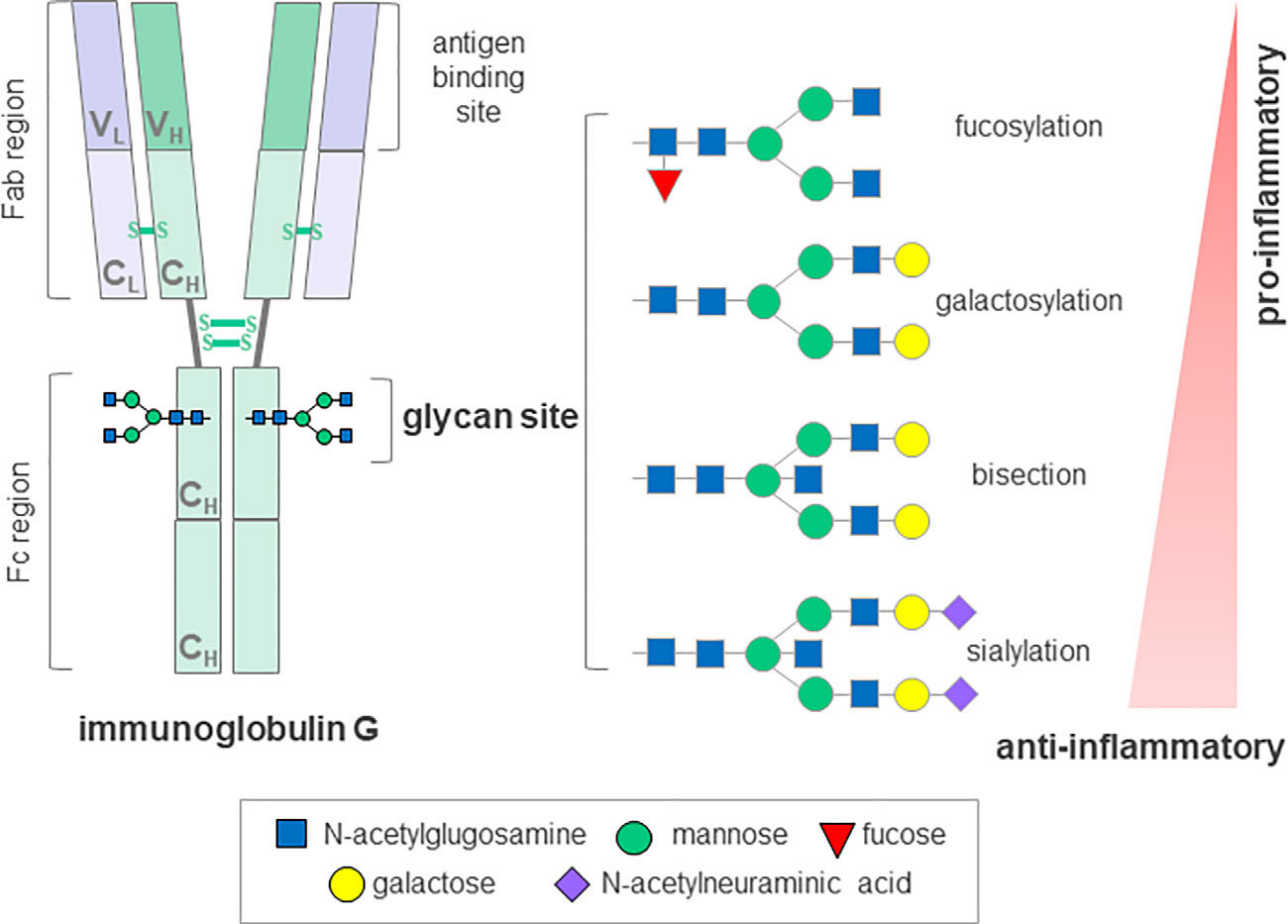
Schematic representation of immunoglobulin G glycosyation. The IgG molecule contains a single conserved glycosylation site on each of its heavy chains that can harbor a variety of glycans. A core glycan structure contains 3 mannoses and 4 N-acetylglucosamines, which can be modified by addition of fucose, bisecting N acetylglucosamine (bisection), galactose and N-acetylneuraminic acid generating up to 30 possible glycan structures. The absence of glycans substantially affects the stability and disrupts the antibody activity. IgG, immunoglobulin G; Fab, antigen-binding fragment; Fc, crystallizable fragment; C; constant domain; V, variable domain; H, heavy chain; L, light chain, S-S, disulfide bond. Depiction according to (67) and (68).

Glycomics is an emerging field, focused on defining and understanding the structures and functions of complex carbohydrates; glycans. Their structural complexity and template-free synthesis involves carefully regulated, dynamic interactions between hundreds of genes, making them the most diverse macromolecules common to all life forms (64, 65). Complex and diverse glycans are ubiquitous to every cell in nature and they play a central role in many key physiological and pathological processes (66). Glycosylation analysis can be performed on the whole serum *N*-glycan profiles as well as on specific glycoproteins, such as immunoglobulin (Ig) G, the most abundant class of antibodies in human plasma. Despite significant heritability of the steady-state composition, IgG glycome composition can change rapidly in the state of pro-inflammatory or anti-inflammatory response (**Figure 2**).

The functional significance of glycans and identification of their biomarker potential in many different human diseases has been facilitated by recent advances in glycomics technologies (69). However, in the area of alloHSCT and its major late complication, cGvHD, glycosylation signatures and mechanisms have been largely unexplored (67).

Aberrant glycosylation has previously been associated with severe immune dysfunction and various autoimmune disorders resembling cGvHD, such as Sjögren’s syndrome, systemic lupus erythematosus, inflammatory bowel disease, rheumatoid arthritis (RA), immune thrombocytopenia, but also with neurological manifestations that can be seen in cGvHD, such as myasthenia gravis, myositis and immune-mediated neuropathies (67, 70). Indeed, distortion of B cell homeostasis, production of autoantibodies and changes in glycosylation of the major effector molecule, IgG, are common to a range of autoimmune diseases (71). The first report on lower IgG galactosylation in patients suffering from RA was published over thirty years ago, and it has since been confirmed in a wide variety of chronic inflammatory and autoimmune diseases, and shown to occur at the level of both total and antigen-specific IgGs. Not only was this change in IgG glycosylation shown to associate with different pathologies, but also with disease progression, activity and severity of symptoms (72). Moreover, in more recent studies on patients suffering from RA, changes in IgG galactosylation levels were described during the remission phase, but also predicted response to therapy and even preceded development of the disease by several years (73–75). Glycans attached to IgG are an integral part of the molecule, influencing its conformation and structural stability as well as its effector and antigen-binding functions (76). It is now well understood that the presence or absence of a specific monosaccharides from IgG glycans can mediate pro- and anti-inflammatory responses, suggesting an active role for differentially glycosylated IgGs in disease development and progression (77) (**Figure 2**). In addition, the finding that glycans affect the inflammatory activity of IgG indicates a promising potential for IgG glycosylation analysis as an additional screening tool to identify individuals with predisposition towards disease development. Furthermore, this involvement in disease pathology could be exploited therapeutically by administration of enzymes, which could modulate IgG glycosylation *in vivo* and hence, modify its activity (78).

Similarly to autoimmune diseases, a higher incidence of circulating autoantibodies has been described in patients with advanced GvHD. However, no confirmation of their pathogenicity, nor association with disease activity or severity has been shown (13). Despite this, extensive examples of the importance of glycosylation and its biomarker potential in other autoimmune settings suggests the considerable potential for glycosylation research in the field of cGvHD, leading to discovery of much needed predictive and/or stratification biomarkers. So far no such evidence has been published, however, a recent study showed altered IgG glycosylation profiles in cGvHD patients compared to healthy controls, and indicated its association with different clinical manifestation of cGvHD, revealing the stratification potential of IgG glycans (79).

While the significance of glycosylation and its manipulation in GvHD processes are slowly being recognized, it is likely that further efforts in this direction could improve monitoring and outcome for patients.

## MicroRNA and cGvHD

MicroRNAs (miRNAs, miRs) are a large family of small (20–22 nucleotides long) non-coding RNAs, which contribute to the control of post-transcriptional gene expression by degrading or suppressing their target mRNA. By regulating target gene signaling pathways, miRNAs influence tumor growth, invasion, metastasis, and angiogenesis. MiRNAs present some characteristics that contribute to their stable expression, such as resistance to RNase, boiling, changes in pH, extended storage, and freeze-thaw cycles (80). Thanks to these aspects, miRNAs can be easily measured using common laboratory techniques. MiRNAs have been shown to present with an altered blood profile in various autoimmune and oncological disorders (81), and thus, they represent a valid option as potential disease biomarkers, including cGvHD.

One of the first reports to implicate miRNAs in cGvHD progression is represented by the study of miR-21 in a cGvHD model of systemic lupus erythematosus (82). Mice deficient in miR-21 presented with a reduction in splenomegaly and a decreased in autoantibodies titers. These effects seemed to be mediated by a skewed Th17 response.

Following this report, interest gathered in other miRNAs in specific GvHD settings. MiR-155 has been found to be upregulated in aGvHD patients, and its expression has been linked not only with the occurrence, but also with the severity of aGvHD (83). Thus, miR-155 is currently under evaluation in a clinical trial assessing its association with the frequency and severity of aGvHD (NCT01521039). In addition, blocking miR-155 expression after alloHSCT has been demonstrated to decrease aGvHD severity and enhance survival in mice (84), thus suggesting its potential use as a novel target therapy in GvHD. After validating its role in aGvHD, miR-155 has been explored in cGvHD settings. A study by Xie et al. (83) showed that miR-155 levels correlate with Th17 and Th9 cell numbers in GvHD patients, promoting Th17 and Th9 cell differentiation in GvHD progression. MiR-155 serum levels are significantly upregulated in cGvHD patients compared to those who did not develop the disease. However, miRNA-155 expression is also significantly elevated in aGvHD patients, and therefore its expression levels do not permit differentiation between the acute and the chronic form of GvHD, excluding its potential function as a cGvHD-specific biomarker.

Recently, miR-17-92 has also been implicated in the pathogenesis of cGvHD. A study by Wu et al. (85) demonstrated that miR-17-92 is crucial for the development of scleroderma and BOS in cGvHD patients *in vivo*. Mechanisms responsible for this role are varied, including the induction of differentiation of pathogenetic Th1 and Th17 cells involved in cGvHD progression. Moreover, miR-17-92 promotes autoantibody production and IgG deposition in the skin of cGvHD patients. Similarly to miR-155, blocking miR-17-19 has been demonstrated to reduce clinical manifestations of cGvHD. These data confirm that a number of miRNAs play a crucial role in cGvHD pathogenesis, suggesting a valid reason to investigate their potential as cGvHD biomarkers. However, no microRNA signature has as yet been identified with a specific blood expression profile in cGvHD patients, and at the moment they cannot be used for monitoring patients or for predicting the risk of developing the disease.

Despite the limited evidence in cGvHD, several miRNAs have been described to identify aGvHD. These include miR-153-3p and miR-29a that have been shown to enhance the risk of aGvHD (86, 87), while miR-146a (88), miR-100 (89), miR-181a (90), and miR-441 (89) are reported to protect patients from the occurrence of GvHD.

Data implicating miRNAs as aGvHD biomarkers supports their study in a cGvHD settings, to identify which miRNAs may be specific for cGvHD and whether they can be effectively used to differentiate between the acute and chronic forms of the disease.

## Extracellular Vesicle Biomarkers for cGvHD

Extracellular vesicles (EVs) are a heterogeneous group of membrane-encapsulated vesicles (40–150 nm in diameter) that derive from the endosomal or plasma membrane (**Table 1**). They are released by most, if not all cell types into the extracellular space and can be sub-classified based on their originating cell and their biogenesis. EVs contain a subset of proteins, lipids and nucleic acids, including microRNA, that are derived from the parent cell (92). It has been shown that EVs have important roles in intercellular communication, both locally and systemically, by transferring their contents between cells. EVs are involved in numerous physiological processes, and vesicles from both non-immune and immune cells have important roles in immune regulation (93).

**Table 1:** General characteristics of extracellular vesicles.

	Exosomes	Microvesicles	Apoptotic Bodies
**Origin**	Endolysosomal pathway; intra-luminal budding of MVB and fusion of MVB with cell membrane	Cell surface; outward budding of cell membrane	Cell surface; outward blebbing of apoptotic cell membrane
**Size**	40–120 nm	50–1,000 nm	500–2,000 nm
**Function**	Intercellular communication	Intercellular communication	Facilitate phagocytosis
**Markers**	Alix, Tsg101, tetraspanins (CD81, CD63, CD9), Flotillin, ESCRT components, annexins	Integrins, selectins, CD40	Annexin V, phosphatidylserine
**Contents**	Nucleic acids (mRNA, miRNA, other non-coding RNAs), cytoplasmic and membrane proteins including receptors and MHC molecules, lipidraft	Nucleic acids (mRNA, miRNA, other non-coding RNAs), cytoplasmic and membrane proteins including receptors, lipidraft	Nuclear fractions, cell organelles

MVB, multivesicular bodies; ESCRT, endosomal sorting complex required for transport; MHC, major histocompatibility complex (91).

The role of EVs in the pathophysiology of immunological disorders is an exciting field for research, as they offer importance as prospective therapeutic targets, informative biological agents and predictive disease biomarkers. Lia et al. (94, 95) investigated a cohort of 41 patients undergoing HSCT for multiple myeloma, in order to explore EV surface marker characterization and any potential correlation with GvHD (aGvHD). A total of 13 EV markers that indicated potential as GvHD biomarkers were assessed by flow cytometry (CD44, CD138, CD146, KRT18, CD120-α, CD8, CD30, CD106, CD25, CD31, CD144, CD86, and CD140-α). Results showed that CD146, CD31 and CD140-α were significantly associated with the onset of aGvHD, and expression of CD146 was associated with an increased aGvHD risk (95). In addition, CD31 fluorescence, CD140-α percentage and CD140-α EV concentration were associated with a decreased risk of aGvHD (95). Finally, CD146 was significantly increased, while CD31 and CD140-α were decreased before the onset of aGvHD (95). While these findings highlight the potential for circulating EVs as biomarkers for GvHD, further investigation is required to investigate the source of EVs, validate the surface biomarker expression and characterise the molecular composition of EVs in order to shed light on their pathological role in aGvHD development. Furthermore, no association was found with cGvHD (94), indicating a distinct role for these EV surface markers in the acute sub-type, which requires further distinction. The role of EV surface markers as biomarkers for the chronic form of GvHD therefore shows potential, but is yet to be defined.

Recently, miRNAs have been identified to be protected from RNAse-mediated degradation in biofluids by encapsulation into EVs (96, 97). Several authors have reported the association of miRNAs with aGvHD in both bio-fluids and within EVs (98–100), thus highlighting the potential for miRNAs as indicative biomarkers in the GvHD setting. However, there are few studies that have focused on chronic forms of the disease or sought to distinguish acute from chronic GvHD.

Crossland et al. (99) identified lower expression of miR-423, miR-199, and miR-93* in the serum EV fraction of patients who subsequently developed aGvHD compared to those who remained GvHD-free. Patients who did not develop GvHD had significantly higher levels of microRNAs within the EV fraction compared to whole serum, and the authors hypothesized that the microRNAs were being specifically transported in the circulation *via* EVs to distal target organs, where they display protective functions (99). However, the authors focused on the acute form of GvHD in their studies and did not extend this work to cGvHD, which would be of particular interest to investigate.

Yoshizawa et al. (100) studied the role of EV miRNA signatures in late onset aGvHD (LA GvHD), with the aim of identifying possible molecular biomarkers. Late onset GvHD is the occurrence of *de novo* aGvHD beyond day-100 post-HSCT, and may comprise 20% to 40% of cGvHD cases according to the 2005 NIH definition (101). Profiling the miRNA cargo of EVs isolated from the plasma of 10 HSCT patients (5 who developed LA GvHD, 5 with no GvHD) and 8 randomly selected healthy controls revealed a signature of 55 microRNAs that were significantly differentially expressed between patients who developed LA GvHD compared to no GvHD, all of which were upregulated in the LA GvHD group (100). The authors selected 10 miRNAs (miR-423-5p, miR-19a, miR-142-3p, miR-128, miR-193b, miR-30c, miR-193a, miR-191, miR-125b, and miR-574-3p) with the highest fold change for validation, of which miR-423-5p, miR-19a, and miR-142 had been previously identified as potential aGvHD biomarkers (99, 102, 103). Expression of miR-423-5p, miR-128, and miR-125b was significantly higher in patients with LA GvHD compared to healthy controls, while miR-423-5p was downregulated in the non-GvHD group compared to controls (100). Assessment of sequential miRNA expression patterns in two HSCT patients revealed that miR-128 was significantly upregulated prior to the onset of LA GvHD, while miR-125 levels were unable to predict LA GvHD, suggesting miR-128 may act as a predictive marker prior to the onset of LA GvHD symptoms (100).

Finally, the most recent, preliminary study of Bogunia-Kubik and colleagues examining the miRNA profile in EVs of cGvHD patients showed differential expression of three miRNAs, miR-630, miR-374b-5p, and miR-29c-3p, in the plasma of patients with cGvHD symptoms compared to samples taken 3 months after transplantation from patients lacking cGvHD (104). Further investigations on larger and well defined patient populations are needed to establish feasible biomarkers extracted from EVs, particularly miRNAs.

## Endothelial-Derived Microparticles and GvHD

While the cytokine storm and T cell activation are the main initiating factors in aGvHD, endothelial damage has also been shown to play an early role, due to endothelial tissue being a primary target of pre-conditioning damage. Cell migration into effector organs requires passage of the endothelial barrier (105). In aGvHD changes in circulating endothelial cell levels are indicated to act as a biomarker for monitoring endothelial damage in patients undergoing HSCT, and may support GvHD diagnosis (106, 107). As a result of the initial pre-conditioning injury, endothelial cell derived microparticles (EMPs) can be released into the circulation, reflecting the extent and possible mechanism of action of endothelial damage independent of T cells. During aGvHD, EMPs can also be released, indicating immune mediated endothelial damage. EMP levels have formed the basis for several studies interested in identifying aGvHD biomarkers and pathological contributors (108–110).

In 2011, Rank et al. investigated plasma levels of erythrocyte-derived microparticles (EryMP) as a potential biomarker to differentiate aGvHD from infection or sepsis (108). They found that plasma levels of EryMP were not affected by total body irradiation, high dose chemotherapy or T cell depletion, nor were they altered in patients who developed infections or sepsis. However, there was a 1.7-fold increase in EryMP levels in patients who developed aGvHD following HSCT compared to pre-HSCT, and also compared to patients who developed infections or sepsis alone. Moreover, the levels of EryMP were associated with the severity of aGvHD (108).

Zhang et al. also focused on EMPs in aGvHD, to further understand the intricate interactions between endothelial cells, T cells and aGvHD (109). They observed significantly higher levels of miR-155 in EMPs of aGvHD patients compared to patients free of aGvHD, whereby miR-155 levels were elevated from 7 days post-transplant and peaked at 28 days post-transplant (109). The group used a TNF-α induced human umbilical vein endothelial cell (HUVEC) injury model to simulate aGvHD *in vitro*, and showed that EMP production was increased, with higher miR-155 levels (109). EMPs were verified to have the ability to act as cell-to-cell communicators, by transferring their cargo to the cytoplasm of T lymphocytes, but delivery of miR-155 was not shown to affect proliferation or apoptosis of the recipient cells (109). With regard to the effect of miR-155 on T cell differentiation, miR-155 inhibition in EMPs suppressed Th1, Th9 and Th17 related cytokines, while Th2-related cytokines were significantly elevated. The frequency of Tregs was also increased, while CD4^+^ T cells were decreased. Collectively, this data suggested that EMPs may mediate the aGvHD response by regulating T cell differentiation towards an aGvHD driving phenotype (109). The same group assessed miR-155 and EMP levels in the peripheral blood of mice during aGvHD and observed similar results to clinical patients, whereby both EMP and miR-155 levels were elevated in aGvHD response, and higher at day +8, +12, and +16 compared to control mice. Interestingly, the group extended their studies by showing that administration of highly concentrated EMPs to aGvHD resulted in increased severity of aGvHD, including enhanced mortality with higher histological aGvHD scores within the liver, spleen and lung. In contrast, in mice receiving miR-155-deficient EMP the manifestation, survival and severity of aGvHD was ameliorated. Furthermore, prevention of endothelial injury following simvastatin administration protected EMP production and improved the overall manifestation and survival of aGvHD mice (109). Collectively, this comprehensive analysis demonstrated a role of miR-155 delivered by EMPs in the initiation or pathogenesis of aGvHD, by activating specific T lymphocytes functions. Thus, highlighting the potential of miR-155 as a therapeutic target for the prevention and treatment of aGvHD, and also as a diagnostic and prognostic surrogate marker (109).

EMPs contain endothelial cell cytoplasmic content as well as RNA and microRNAs, and are able to transfer this content between cells. Niek et al. hypothesized that in a transplant setting, EMPs may transport Hedgehog-interacting protein (HIP), a transmembrane glycoprotein abundantly expressed in endothelial cells, to target endothelial cells for aggravated damage (110). HIPP is an inhibitor of the Sonic hedgehog (SHH) signaling pathway (111), which functions to reduce endothelial cells apoptosis (112). Collectively, this may result in sustained endothelial damage during aGvHD development (110). Given the relevance of endothelial damage in cGvHD, it would be of great interest to investigate EMPs as a biomarker for the chronic form of the disease, however, distinction from aGvHD as well as late onset subtypes remains a challenge.

## DNA Methylation and cGvHD

**Figure 3:**
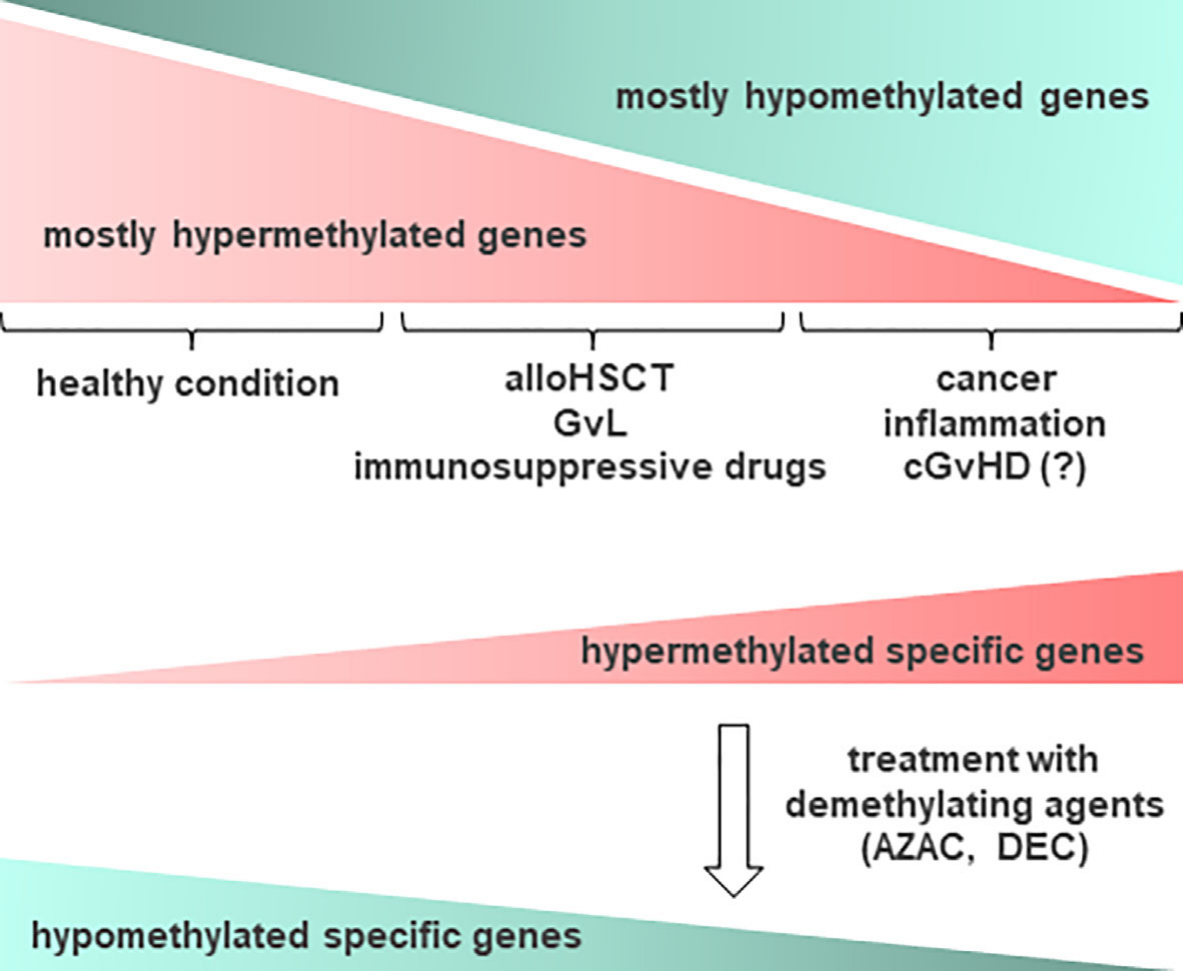
Schematic representation of DNA methylation occurrence in relation to cGvHD and other conditions. In healthy condition, epigenetic changes in DNA methylation occur often on most of cellular gene promoters, resulting in the absence of gene expression. In cancer, inflammation and other conditions such as cGvHD, some relevant genes become methylated and as such, suppressed in their function. Accordingly, aberrant DNA methylation profiles can point to the diseased tissue, providing important information prior to treatment. Epigenetic drugs, such as demethylating agents (DNA methyltransferase (DNMT) inhibitors), for example azacytidine (AZAC) and decitabine (DEC), may be used to induce expression of certain genes, particularly after allogeneic hematopoietic stem cell transplantation (alloHSCT), enhancing the graft versus leukemia (GvL) effect. Thus, demethylating agents could be considered a therapy of choice for recovering from cGvHD. Hypermethylated genes are presented in red, while hypomethylated in green color.

Aberrant DNA methylation is a key pathological mechanism in myelodysplastic syndromes (MDS) and acute myeloid leukemia (AML). This provides rationale for the clinical development of epigenetic regulators, hypomethylating agents (HMAs), for example azacytidine (AZAC) and decitabine (DEC), for the treatment of these diseases (**Figure 3**). Studies have shown that AZAC after alloHSCT increases regulatory T cell (Treg) numbers while inducing a cytotoxic CD8+ T cell response, suggesting a potential mechanism for augmenting the graft versus leukemia (GvL) effect without increasing GvHD. In patients at a high risk of relapse following alloHSCT, pre-emptive AZAC may help prevent or delay relapse. For patients who have relapsed following alloHSCT, AZAC may be a salvage therapy option, either as monotherapy or in combination with donor lymphocyte infusions (DLI) (113).

It is known that human leukocyte antigen-G (HLA-G) expression is strongly regulated by DNA methylation. In the study of Stamou et al. the authors investigated whether HMAs, such as AZAC and DEC, may be used to induce HLA-G expression on conventional T cells and convert them to Tregs. HMAs treatment induced *de novo* expression of HLA-G on T cells through hypomethylation of the HLA-G proximal promoter. The HMA-induced CD4+HLA-Gpos T cells are Foxp3 negative and have potent *in vitro* suppression function, which is dependent to a large extent, but not exclusively, on the HLA-G molecule. Converted HLA-Gpos suppressors retain their suppressor function in the presence of tumor necrosis factor (TNF) and preserve hypomethylated the HLA-G promoter for at least 2 days after AZAC exposure. DEC-treated T cells suppressed *ex vivo* the proliferation of T cells isolated from patients suffering from GvHD (114). HMAs have an advantage over conventional cytotoxic chemotherapeutic agents in terms of regimen-related toxicity in high-risk patients. With regard to transplantation, recent studies have suggested that HMAs may potentiate GvL effects. Therefore, a pilot study of AZAC treatment after discontinuation of immunosuppressants was performed in MDS patients who relapsed after transplantation. There was no association between the treatment response and emergence of cGvHD. Because all patients discontinued immunosuppressants, it was impossible to determine whether AZAC potentiated the GvL effects. Taking into consideration the limitation of the small number of cases reported in the pilot study, the results suggest that treatment of patients with MDS, after relapse post-transplantation, with HMAs is feasible and the additional GvL effects might signiﬁcantly affect patient outcome (115).

In the study of Czibere et al. it is clear that treatment with the DNA methylation inhibitor, 5-azacytidine (5-AZAC), has an inﬂuence on the donor immune system. Moreover, previous reports showed induction of killer Ig-like receptors in natural killer (NK) cells following treatment with DNA demethylating agents. It is well established that NK cells are involved in the speciﬁc recognition of leukemic target cells and can enhance the GvL effect. Thus, they hypothesized that an interplay between 5-AZAC and DLI, in which treatment with 5-AZAC ﬁrst, leads to a reduction of the leukemic burden by an anti-proliferative effect on the leukemic target cells and then modulates the donor T and NK cell compartment. This might be able to consolidate 5-AZAC-induced complete resolution of GvHD manifestations in all organs (116).

The epigenotype of myeloid leukemic blasts, as for other neoplasias, is marked by large sets of genes being heavily repressed through DNA methylation and inactivating histone modiﬁcations. These include tumor suppressor genes, antiapoptotic and cell-cycle regulatory genes. The pharmaceutical industry is developing treatment approaches in leukemia and MDS using hypomethylating agents, which revert aberrant DNA hypermethylation, and histone deacetylase inhibitors that antagonize promoter silencing. Although hypomethylating agents have shown very interesting activity in large phase II and III clinical trials of MDS, different combinations of these drugs with other therapeutics in leukemia and MDS are under consideration. The optimal dose of HMA has not yet been established, because hematopoiesis may be much more sensitive to the myelotoxicity mediated sometimes by even low doses of the drugs. Because of the previous studies with promising activity of a 3-day schedule of DEC, and the effectiveness of a 3-day schedule of low-dose 5-AZAC in reinducing fetal hemoglobin, herein they have embarked on a sequential treatment of AML/high risk MDS patients with 5-AZAC given for 3 days followed by DLI. This treatment showed promising feasibility, with a very low GvHD rate, and clinical beneﬁt in 66% of the patients, including attainment of continued CR in four patients in whom previous DLI alone had not been sufﬁcient for disease control. The triggering of GvHD by the sequence of 5-AZAC and DLI treatment is a serious theoretical concern, as the DNA demethylating agents could hypothetically upregulate antigens also on normal, bystander tissue such as skin, gut and liver. Thus, the low rate of *de novo* GvHD in this patient series was very encouraging, arguing against an off-target effect of this HMA on normal cells, at least at the dose and schedule given. In conclusion, these results are supportive of a model in which the effect of active immunotherapy can be boosted by an epigenetically active drug (117).

In summary, analyses of DNA methylomes, and in combination with microRNAs and RNA transcriptomes with high-throughput methods could give insight into the epigenetic background of cGvHD symptoms and greatly assist in developing and evaluating new approaches to its treatment.

## Microbiome and cGvHD

Whereas the impact of microbiota on aGvHD after alloHSCT has been intensively investigated in the literature, the knowledge of microbes relating to the pathophysiology of cGvHD is highly restricted. However, loss of intestinal microbiome diversity and a shift towards an enteropathogeneic flora has been observed during alloHSCT, mainly due to the use of systemic antibiotics for prophylaxis and treatment of neutropenic infections, conditioning toxicity and an altered food intake. In particular, a reduction of commensal bacteria like *Clostridiales bacterium* and their protective metabolites *e.g.* indoles and short chain fatty acids have been reported to enhance the risk for a disturbed immune tolerance, development of GvHD and high transplant-related mortality after HSCT (118–120). Moreover, to which extent microbial changes also contribute to the development of cGvHD is poorly evaluated; only a few groups have investigated microbial changes in cGvHD patients so far.

Shimizu et al. (121) explored the status of microbes in the ocular surface in patients with ocular cGvHD and discovered alterations in the composition of ocular surface microbes. Approximately fifty percent of alloHSCT recipients developed dry eye syndrome, the main phenotype of ocular cGvHD within 2 years after alloHSCT. Against expectations, these patients showed a more complex diversity of ocular surface microbes compared with nonGvHD patients and healthy controls. The majority of detected species were *Staphylococcus epidermidis*, *Corynebacterium* species, and *Propionibacterium* acnes. In fact, these strains display commensal bacteria of the ocular surface, but their detection ratio was significantly higher in GvHD patients and associated with an increased risk for GvHD development compared to patients without GvHD and healthy controls. Pathogenic bacteria such as α-hemolitic *Streptococcus*, *Haemophilus influenza*, and *Enterobacter cloacae* were also detected more frequently in patients suffering from ocular cGvHD. These results suggest that alternations of microbes may be involved in the pathogenic process of ocular cGvHD (121). A potential explanation for the broader bacterial diversity in ocular cGvHD may be the loss of protective factors, including secretory immunoglobulin A (SIgA) and defensins, as well as alteration of the quantity of tear fluid.

There is clear evidence that the microbiome has potent immunoregulatory functions (122). The association between changes of microbiota composition and the development of immune-mediated disorders has been extensively investigated and it becomes increasingly evident that microbial dysbiosis acts as an important contributor to the pathophysiology of various immune diseases, such as inflammatory bowel disease, Sjögren’s syndrome, autoimmune hepatitis, rheumatoid arthritis, systemic sclerosis, etc. (123, 124). De Paiva et al. (122) assessed the effects of intestinal dysbiosis in a murine model and characterized the conjunctival, tongue and fecal microbiome profiles of patients with SS. They observed a clear inverse correlation between severity of ocular and systemic sclerosis and intestinal microbial diversity, assuming that it is driven by low relative abundance of commensal bacteria like *Clostridium* and high relative abundance of potentially pathogenic genera e.g. *Enterobacter*, *Escherichia*, *Shigella* and *Pseudomonas* (122). Increased intestinal permeability, derangement of the microbiome and bacterial translocation have also been described in experimental and human AIH, correlating with the severity of the disease (125). Emerging studies have demonstrated that the role of the gut and lung microbiota and the resulting immune engagement cannot be ignored when studying the pathophysiology of chronic lung diseases (126). Several studies also suggest a link between microbiota composition of the lower respiratory tract and also the gut and respiratory health in patients suffering from cystic fibrosis and BOS, the primary limiting factor for long-term survival after lung transplantation. Lung transplant patients have a unique lung microbiome that is different compared to the pretransplanted microbiome (127). Re-establishment of pretransplant lung microbial populations in the alloSCT seems to have a protective effect against BOS, whereas *de novo* acquisition of microbial populations may increase the risk (128). BOS is also a serious pulmonary complication leading to late morbidity and decreased survival after alloSCT.

Hildebrandt et al. (129) demonstrated an important role for recipient NOD2/CARD15 gene variants as an independent risk factor for BOS development. NOD2/CARD15 displays an intracellular receptor for bacterial cell wall products expressed in epithelial cells of the intestinal tract, in bronchial epithelium, in macrophages, monocytes, and antigen-presenting cells. NOD2/CARD15 mutations may lead to an altered microbial composition resulting in an increased susceptibility to subclinical pulmonary infections, dysregulated and prolonged inflammation, bronchial epithelial injury and finally airway obstruction (129). These findings could be recently confirmed by Touihri et al. (130) in a group of Tunisian ASCT recipients.

A variety of further studies demonstrate associations between microbiome dysbiosis and immune diseases. In addition, the impact of the virome on the pathophysiology of immune-mediated diseases and cGvHD should not be underestimated. For instance, cytomegalovirus seropositivity of the recipient and the donor prior to alloHSCT was observed as a risk factor for the development of cGvHD (131). New data exist showing that phages have anti-inflammatory and immunomodulatory activities, an observation that may be translated into a novel treatment of GvHD and may stimulate new directions of research (132).

In summary, dysbiosis of gut, oral, and skin microbiome has been linked to auto-inflammation and tissue damage although precise mechanistic links that remain largely unknown (124). The pathophysiology of cGvHD and many autoimmune disorders is very similar, suggesting the assumption that microbiota may also play a contributory role in the development of cGvHD. In the acute form of GvHD specific bacterial strains and also microbial metabolites are considered as potential biomarkers for intestinal dysbiosis. However, the current literature lacks detailed high-level studies evaluating the role of microbiota in the chronic form of GvHD and microbial biomarkers so far. Further clinical and experimental studies are required to illuminate the impact of microbiota in the pathophysiology of cGvHD.

## Conclusion

A multidisciplinary approach is needed to better understand the chronic form of GvHD. Thus in this review, we attempted to provide a comprehensive description of potential novel diagnostic, prognostic or predictive biomarkers for cGvHD (**Table 2**). An evolving signature of potential biomarkers should be considered for future evaluation in well-defined clinical trials. For example, while it is clear that a high level of donor-specific-antibodies is a risk factor for HSCT transplantation, the degree to which further characterization or further identification of DSA would be helpful remains to be determined. MicroRNAs and extracellular vesicle-derived RNAs are an exciting novel field for cGvHD biomarker discovery, and have recently gained increasing interest in relation to HSCT outcome and aGvHD complications. Their further exploration holds great potential, due to their many advantages as clinically translatable biomarkers. In addition, DNA methylomes and microRNAs, together with RNA transcriptome analysis could give better insight into the epigenetic background of cGvHD as well as patients’ follow-up. As potential novel biomarkers, epigenetic tools could considerably assist in the development of new treatment approaches, particularly introducing epigenetic therapy combined with immunotherapy. Similarly, IgG glycan profiling offers a novel tool for cGvHD staging and possibly prediction, but needs further investigation. Finally, while the microbiome seems to be important in aGvHD after alloHSCT, its role in the pathophysiology of cGvHD, although probably important, has to be explored.

**Table 2:** Highlights.

There are potential novel and still unexplored biomarkers associated with cGvHD.
Biomarkers of either diagnostic, prognostic or predictive value are essential for assessment and monitoring of cGvHD.
Candidate cellular biomarkers have been identified for cGvHD but need further validation.
Both host-reactive antibodies in general, and particularly alloantibodies have been associated with cGvHD.
Glycans that are directly involved in the pathophysiology of every major disease have a great research potential in clarifying events after alloHSCT and GvHD.
Epigenetic changes such as microRNAs and DNA methylation represent potential biomarkers for monitoring cGvHD patients and for developing new treatment approaches.
Extracellular vesicles, essential in the regulation of numerous physiological processes in both non-immune and immune cells, represent an excellent source of biological material for biomarker investigation.
Endothelial derived microparticles reflect the extent of endothelial damage occurring during aGvHD and have merit for exploration in a cGvHD setting.
The microbiome likely affects the pathophysiology of cGvHD.

## Author Contributions

MG designed and coordinated the preparation of this manuscript. HG wrote the section on cellular biomarkers for cGvHD. NM and KBK wrote the section on alloantibodies and GvHD of the manuscript. FP and RC wrote the section on microRNA and cGvHD of the manuscript. RC, FP, and KBK wrote the sections on extracellular vesicle and cGvHD, as well as the endothelial-derived microparticles and cGvHD of the manuscript. NG wrote the section on DNA methylation and cGvHD of the manuscript. MB and DP wrote the section on glycomics in cGvHD of the manuscript. DWe, EH, and DWo wrote the section on microbiome and cGvHD of the manuscript; AD and MI contributed to the final version of the manuscript. All authors contributed to the article and approved the submitted version.

## Funding

This work was supported by the European Cooperation in Science & Technology under the COST Action CA17138 (Integrated European Network on Chronic Graft Versus Host Disease: EUROGRAFT; https://www.gvhd.eu) initiated and chaired by AMD from Newcastle University, UK, and further coordinated as grant holder by MI from Oslo University Hospital Oslo, Norway. KBK is, in addition, supported by the grant no. 2018/31/B/NZ2/03065 from the National Science Centre (Poland). NM was supported by Vaincre la Mucoviscidose (CAP 2020) and the INSERM (France). REC is supported by the Newcastle upon Tyne Hospitals NHS Charity. MB and DP were supported by the Croatian Science Foundation Project IP-2016-06-8046 (New biomarkers for chronic graft-versus-host disease). DWo was supported by the Deutsche Forschungsgemeinschaft (DFG, German Research Foundation), Project ID 324392634 - TRR221.

## Conflict of Interest

The authors declare that the research was conducted in the absence of any commercial or financial relationships that could be construed as a potential conflict of interest.

The reviewer BB declared a shared affiliation, with no collaboration, with one of the authors, FP, to the handling editor at the time of review.

## References

[1] Zeiser R Blazar BR Pathophysiology of Chronic Graft-versus-Host Disease and Therapeutic Targets. N Engl J Med (2017) 377:2565–79. 10.1056/NEJMra1703472 29281578

[2] Bôle-Richard E Gamonet C Certoux J-M Idirene I Larosa F Deconinck E Exposure to hypomethylating agent, 5-azacytidine, may improve iCasp9 suicide gene therapy for treating GvHD in allografts. Gene Ther (2016) 23:664–72. 10.1038/gt.2016.39 27111151

[3] Jagasia MH Greinix HT Arora M Williams KM Wolff D Cowen EW National Institutes of Health Consensus Development Project on Criteria for Clinical Trials in Chronic Graft-versus-Host Disease: I. The 2014 Diagnosis and Staging Working Group report. Biol Blood Marrow Transplant (2015) 21:389–401.e1. 10.1016/j.bbmt.2014.12.001 25529383 PMC4329079

[4] Wolff D Greinix H Lee SJ Gooley T Paczesny S Pavletic S Biomarkers in chronic graft-versus-host disease: quo vadis? Bone Marrow Transplant (2018) 53:832–7. 10.1038/s41409-018-0092-x PMC6041126 29367715

[5] Partanen J Hyvärinen K Bickeböller H Bogunia-Kubik K Crossland RE Ivanova M Review of Genetic Variation as a Predictive Biomarker for Chronic Graft-Versus-Host-Disease After Allogeneic Stem Cell Transplantation. Front Immunol (2020) 11:575492. 10.3389/fimmu.2020.575492 33193367 PMC7604383

[6] Cooke KR Luznik L Sarantopoulos S Hakim FT Jagasia M Fowler DH The Biology of Chronic Graft-versus-Host Disease: A Task Force Report from the National Institutes of Health Consensus Development Project on Criteria for Clinical Trials in Chronic Graft-versus-Host Disease. Biol Blood Marrow Transplant (2017) 23:211–34. 10.1016/j.bbmt.2016.09.023 PMC6020045 27713092

[7] Paczesny S Hakim FT Pidala J Cooke KR Lathrop J Griffith LM National Institutes of Health Consensus Development Project on Criteria for Clinical Trials in Chronic Graft-versus-Host Disease: III. The 2014 Biomarker Working Group Report. Biol Blood Marrow Transplant (2015) 21:780–92. 10.1016/j.bbmt.2015.01.003 PMC4408233 25644957

[8] Sarantopoulos S Stevenson KE Kim HT Cutler CS Bhuiya NS Schowalter M Altered B-cell homeostasis and excess BAFF in human chronic graft-versus-host disease. Blood (2009) 113:3865–74. 10.1182/blood-2008-09-177840 PMC2670799 19168788

[9] Allen JL Fore MS Wooten J Roehrs PA Bhuiya NS Hoffert T B cells from patients with chronic GVHD are activated and primed for survival via BAFF-mediated pathways. Blood (2012) 120:2529–36. 10.1182/blood-2012-06-438911 PMC3448264 22896003

[10] Allen JL Tata PV Fore MS Wooten J Rudra S Deal AM Increased BCR responsiveness in B cells from patients with chronic GVHD. Blood (2014) 123:2108–15. 10.1182/blood-2013-10-533562 PMC3968393 24532806

[11] Flynn R Allen JL Luznik L MacDonald KP Paz K Alexander KA Targeting Syk-activated B cells in murine and human chronic graft-versus-host disease. Blood (2015) 125:4085–94. 10.1182/blood-2014-08-595470 PMC4481596 25852057

[12] Greinix HT Pohlreich D Kouba M Körmöczi U Lohmann I Feldmann K Elevated Numbers of Immature/Transitional CD21– B Lymphocytes and Deficiency of Memory CD27+ B Cells Identify Patients with Active Chronic Graft-versus-Host Disease. Biol Blood Marrow Transplant (2008) 14:208–19. 10.1016/j.bbmt.2007.10.009 18215781

[13] Kuzmina Z Greinix HT Weigl R Körmöczi U Rottal A Frantal S Significant differences in B-cell subpopulations characterize patients with chronic graft-versus-host disease-associated dysgammaglobulinemia. Blood (2011) 117:2265–74. 10.1182/blood-2010-07-295766 21063025

[14] Kuzmina Z Krenn K Petkov V Körmöczi U Weigl R Rottal A CD19+CD21low B cells and patients at risk for NIH-defined chronic graft-versus-host disease with bronchiolitis obliterans syndrome. Blood (2013) 121:1886–95. 10.1182/blood-2012-06-435008 23303823

[15] Greinix HT Kuzmina Z Weigl R Körmoczi U Rottal A Wolff D CD19+CD21low B Cells and CD4+CD45RA+CD31+ T Cells Correlate with First Diagnosis of Chronic Graft-versus-Host Disease. Biol Blood Marrow Transplant (2015) 21:250–8. 10.1016/j.bbmt.2014.11.010 25460358

[16] She K Gilman AL Aslanian S Shimizu H Krailo M Chen Z Altered Toll-Like Receptor 9 Responses in Circulating B Cells at the Onset of Extensive Chronic Graft-versus-Host Disease. Biol Blood Marrow Transplant (2007) 13:386–97. 10.1016/j.bbmt.2006.12.441 17382246

[17] Rosser EC Mauri C Regulatory B Cells: Origin, Phenotype, and Function. Immunity (2015) 42:607–12. 10.1016/j.immuni.2015.04.005 25902480

[18] Khoder A Sarvaria A Alsuliman A Chew C Sekine T Cooper N Regulatory B cells are enriched within the IgM memory and transitional subsets in healthy donors but are deficient in chronic GVHD. Blood (2014) 124:2034–45. 10.1182/blood-2014-04-571125 PMC4186534 25051962

[19] de Masson A Bouaziz J-D Le Buanec H Robin M O’Meara A Parquet N CD24hiCD27+ and plasmablast-like regulatory B cells in human chronic graft-versus-host disease. Blood (2015) 125:1830–9. 10.1182/blood-2014-09-599159 25605369

[20] Zorn E Kim HT Lee SJ Floyd BH Litsa D Arumugarajah S Reduced frequency of FOXP3+ CD4+CD25+ regulatory T cells in patients with chronic graft-versus-host disease. Blood (2005) 106:2903–11. 10.1182/blood-2005-03-1257 PMC1895303 15972448

[21] Clave E Busson M Douay C Peffault de Latour R Berrou J Rabian C Acute graft-versus-host disease transiently impairs thymic output in young patients after allogeneic hematopoietic stem cell transplantation. Blood (2009) 113:6477–84. 10.1182/blood-2008-09-176594 19258596

[22] Matsuoka K Kim HT McDonough S Bascug G Warshauer B Koreth J Altered regulatory T cell homeostasis in patients with CD4+ lymphopenia following allogeneic hematopoietic stem cell transplantation. J Clin Invest (2010) 120:1479–93. 10.1172/JCI41072 PMC2860902 20389017

[23] Chen X Vodanovic-Jankovic S Johnson B Keller M Komorowski R Drobyski WR Absence of regulatory T-cell control of TH1 and TH17 cells is responsible for the autoimmune-mediated pathology in chronic graft-versus-host disease. Blood (2007) 110:3804–13. 10.1182/blood-2007-05-091074 PMC2077325 17693581

[24] Hill GR Olver SD Kuns RD Varelias A Raffelt NC Don AL Stem cell mobilization with G-CSF induces type 17 differentiation and promotes scleroderma. Blood (2010) 116:819–28. 10.1182/blood-2009-11-256495 20435882

[25] Dander E Balduzzi A Zappa G Lucchini G Perseghin P Andrè V Interleukin-17–Producing T-Helper Cells as New Potential Player Mediating Graft-Versus-Host Disease in Patients Undergoing Allogeneic Stem-Cell Transplantation. Transplantation (2009) 88:1261–72. 10.1097/TP.0b013e3181bc267e 19996925

[26] Brüggen M-C Klein I Greinix H Bauer W Kuzmina Z Rabitsch W Diverse T-cell responses characterize the different manifestations of cutaneous graft-versus-host disease. Blood (2014) 123:290–9. 10.1182/blood-2013-07-514372 24255916

[27] Imanguli MM Swaim WD League SC Gress RE Pavletic SZ Hakim FT Increased T-bet+ cytotoxic effectors and type I interferon–mediated processes in chronic graft-versus-host disease of the oral mucosa. Blood (2009) 113:3620–30. 10.1182/blood-2008-07-168351 PMC2668847 19168793

[28] Forcade E Paz K Flynn R Griesenauer B Amet T Li W An activated Th17-prone T cell subset involved in chronic graft-versus-host disease sensitive to pharmacological inhibition. JCI Insight (2017) 2:e92111. 10.1172/jci.insight.92111 PMC5470889 28614794

[29] Forcade E Kim HT Cutler C Wang K Alho AC Nikiforow S Circulating T follicular helper cells with increased function during chronic graft-versus-host disease. Blood (2016) 127:2489–97. 10.1182/blood-2015-12-688895 PMC4874229 26944544

[30] Kariminia A Holtan SG Ivison S Rozmus J Hebert M-J Martin PJ Heterogeneity of chronic graft-versus-host disease biomarkers: association with CXCL10 and CXCR3+ NK cells. Blood (2016) 127:3082–91. 10.1182/blood-2015-09-668251 PMC4911864 27020088

[31] Schultz KR Kariminia A Ng B Abdossamadi S Lauener M Nemecek ER Immune profile differences between chronic GVHD and late acute GVHD: results of the ABLE/PBMTC 1202 studies. Blood (2020) 135:1287–98. 10.1182/blood.2019003186 PMC7146024 32047896

[32] Loupy A Lefaucheur C Vernerey D Prugger C van Huyen J-PD Mooney N Complement-Binding Anti-HLA Antibodies and Kidney-Allograft Survival. N Engl J Med (2013) 369:1215–26. 10.1056/NEJMoa1302506 24066742

[33] Lepin EJ Zhang Q Zhang X Jindra PT Hong LS Ayele P Phosphorylated S6 Ribosomal Protein: A Novel Biomarker of Antibody-Mediated Rejection in Heart Allografts. Am J Transplant (2006) 6:1560–71. 10.1111/j.1600-6143.2006.01355.x 16827856

[34] Jin Y-P Valenzuela NM Zhang X Rozengurt E Reed EF HLA Class II–Triggered Signaling Cascades Cause Endothelial Cell Proliferation and Migration: Relevance to Antibody-Mediated Transplant Rejection. J Immunol (2018) 200:2372–90. 10.4049/jimmunol.1701259 PMC5860975 29475988

[35] Lion J Taflin C Cross AR Robledo-Sarmiento M Mariotto E Savenay A HLA Class II Antibody Activation of Endothelial Cells Promotes Th17 and Disrupts Regulatory T Lymphocyte Expansion. Am J Transplant (2016) 16:1408–20. 10.1111/ajt.13644 26614587

[36] Muller TF Kraus M Neumann C Lange H Detection of renal allograft rejection by complement components C5A and TCC in plasma and urine. J Lab Clin Med (1997) 129:62–71. 10.1016/S0022-2143(97)90162-1 9011592

[37] Ruggeri A Rocha V Masson E Labopin M Cunha R Absi L Impact of donor-specific anti-HLA antibodies on graft failure and survival after reduced intensity conditioning-unrelated cord blood transplantation: a Eurocord, Société Francophone d’Histocompatibilité et d’Immunogénétique (SFHI) and Société Française de Greffe de Moelle et de Thérapie Cellulaire (SFGM-TC) analysis. Haematologica (2013) 98:1154–60. 10.3324/haematol.2012.077685 PMC3696621 23242594

[38] Ciurea SO Thall PF Milton DR Barnes TH Kongtim P Carmazzi Y Complement-Binding Donor-Specific Anti-HLA Antibodies and Risk of Primary Graft Failure in Hematopoietic Stem Cell Transplantation. Biol Blood Marrow Transplant (2015) 21:1392–8. 10.1016/j.bbmt.2015.05.001 PMC4506716 25985919

[39] Loren AW Bunin GR Boudreau C Champlin RE Cnaan A Horowitz MM Impact of Donor and Recipient Sex and Parity on Outcomes of HLA-Identical Sibling Allogeneic Hematopoietic Stem Cell Transplantation. Biol Blood Marrow Transplant (2006) 12:758–69. 10.1016/j.bbmt.2006.03.015 16785065

[40] El-Awar N Terasaki PI Nguyen A Sasaki N Morales-Buenrostro LE Saji H Epitopes of human leukocyte antigen class I antibodies found in sera of normal healthy males and cord blood. Hum Immunol (2009) 70:844–53. 10.1016/j.humimm.2009.06.020 19580837

[41] Morales-Buenrostro LE Terasaki PI Marino-Vázquez LA Lee J-H El-Awar N Alberú J “Natural” Human Leukocyte Antigen Antibodies Found in Nonalloimmunized Healthy Males. Transplantation (2008) 86:1111–5. 10.1097/TP.0b013e318186d87b 18946350

[42] Ciurea SO Thall PF Wang X Wang SA Hu Y Cano P Donor-specific anti-HLA Abs and graft failure in matched unrelated donor hematopoietic stem cell transplantation. Blood (2011) 118:5957–64. 10.1182/blood-2011-06-362111 PMC3761379 21967975

[43] Gladstone DE Zachary AA Fuchs EJ Luznik L Kasamon YL King KE Partially Mismatched Transplantation and Human Leukocyte Antigen Donor-Specific Antibodies. Biol Blood Marrow Transplant (2013) 19:647–52. 10.1016/j.bbmt.2013.01.016 PMC3768172 23353119

[44] Srinivasan M Flynn R Price A Ranger A Browning JL Taylor PA Donor B-cell alloantibody deposition and germinal center formation are required for the development of murine chronic GVHD and bronchiolitis obliterans. Blood (2012) 119:1570–80. 10.1182/blood-2011-07-364414 PMC3286218 22072556

[45] Spellman S Bray R Rosen-Bronson S Haagenson M Klein J Flesch S The detection of donor-directed, HLA-specific alloantibodies in recipients of unrelated hematopoietic cell transplantation is predictive of graft failure. Blood (2010) 115:2704–8. 10.1182/blood-2009-09-244525 PMC2852369 20089963

[46] Bogunia-Kubik K Łacina P From genetic single candidate gene studies to complex genomics of GvHD. Br J Haematol (2017) 178:661–75. 10.1111/bjh.14704 28444735

[47] Delbos F Barhoumi W Cabanne L Beckerich F Robin C Redjoul R Donor Immunization Against Human Leukocyte Class II Antigens is a Risk Factor for Graft-versus-Host Disease. Biol Blood Marrow Transplant (2016) 22:292–9. 10.1016/j.bbmt.2015.09.027 26453972

[48] Pan Z Yuan X Li Y Wu X Zhu W Bao X Dynamic Detection of Anti–Human Leukocyte Antigen (HLA) Antibodies but not HLA-DP Loci Mismatches Can Predict Acute Graft-versus-Host Disease and Overall Survival in HLA 12/12–Matched Unrelated Donor Allogeneic Hematopoietic Stem Cell Transplantation for Hematological Malignancies. Biol Blood Marrow Transplant (2016) 22:86–95. 10.1016/j.bbmt.2015.08.015 26283096

[49] Takanashi M Atsuta Y Fujiwara K Kodo H Kai S Sato H The impact of anti-HLA antibodies on unrelated cord blood transplantations. Blood (2010) 116:2839–46. 10.1182/blood-2009-10-249219 20628152

[50] Zou J Romee R Slade M Phelan D Keller J Mohanakumar T Untreated donor specific antibodies against HLA are associated with poor outcomes in peripheral blood haploidentical hematopoietic cell transplantation. Bone Marrow Transplant (2017) 52:898–901. 10.1038/bmt.2017.7 28218756 PMC6298599

[51] Yoshihara S Maruya E Taniguchi K Kaida K Kato R Inoue T Risk and prevention of graft failure in patients with preexisting donor-specific HLA antibodies undergoing unmanipulated haploidentical SCT. Bone Marrow Transplant (2012) 47:508–15. 10.1038/bmt.2011.131 21691261

[52] Drénou B Blancheteau V Burgess DH Fauchet R Charron DJ Mooney NA A Caspase-Independent Pathway of MHC Class II Antigen-Mediated Apoptosis of Human B Lymphocytes. J Immunol (1999) 163:4115–24. 10510346

[53] Carmagnat M Drénou B Chahal H Lord JM Charron D Estaquier J Dissociation of caspase-mediated events and programmed cell death induced via HLA-DR in follicular lymphoma. Oncogene (2006) 25:1914–21. 10.1038/sj.onc.1209222 16301998

[54] Ivanov A Beers SA Walshe CA Honeychurch J Alduaij W Cox KL Monoclonal antibodies directed to CD20 and HLA-DR can elicit homotypic adhesion followed by lysosome-mediated cell death in human lymphoma and leukemia cells. J Clin Invest (2009) 119:2143–59. 10.1172/JCI37884 PMC2719942 19620786

[55] Aljabri A Vijayan V Stankov M Nikolin C Figueiredo C Blasczyk R HLA class II antibodies induce necrotic cell death in human endothelial cells via a lysosomal membrane permeabilization-mediated pathway. Cell Death Dis (2019) 10:1–15. 10.1038/s41419-019-1319-5 PMC6408495 30850581

[56] Cross AR Lion J Loiseau P Charron D Taupin J-L Glotz D Donor Specific Antibodies are not only directed against HLA-DR: Minding your Ps and Qs. Hum Immunol (2016) 77:1092–100. 10.1016/j.humimm.2016.04.003 27060781

[57] Cross AR Lion J Poussin K Assayag M Taupin J-L Glotz D HLA-DQ alloantibodies directly activate the endothelium and compromise differentiation of FoxP3high regulatory T lymphocytes. Kidney Int (2019) 96:689–98. 10.1016/j.kint.2019.04.023 31307777

[58] Senev A Lerut E Sandt VV Coemans M Callemeyn J Sprangers B Specificity, strength, and evolution of pretransplant donor-specific HLA antibodies determine outcome after kidney transplantation. Am J Transplant (2019) 19:3100–13. 10.1111/ajt.15414 31062492

[59] Iwaszko M Bogunia-Kubik K Clinical Significance of the HLA-E and CD94/NKG2 Interaction. Arch Immunol Ther Exp (2011) 59:353. 10.1007/s00005-011-0137-y 21800130

[60] Crocchiolo R Ringden O Bay J-O Blaise D Omasic B Mazzi B Impact of HLA-G polymorphism on the outcome of allogeneic hematopoietic stem cell transplantation for metastatic renal cell carcinoma. Bone Marrow Transplant (2018) 53:213–8. 10.1038/bmt.2017.243 29131154

[61] Miklos DB Kim HT Miller KH Guo L Zorn E Lee SJ Antibody responses to H-Y minor histocompatibility antigens correlate with chronic graft-versus-host disease and disease remission. Blood (2005) 105:2973–8. 10.1182/blood-2004-09-3660 PMC1350982 15613541

[62] Lefaucheur C Viglietti D Bouatou Y Philippe A Pievani D Aubert O Non-HLA agonistic anti-angiotensin II type 1 receptor antibodies induce a distinctive phenotype of antibody-mediated rejection in kidney transplant recipients. Kidney Int (2019) 96:189–201. 10.1016/j.kint.2019.01.030 31005275

[63] Dragun D Fritsche L Kintscher U Eckert D Schönemann C Neumayer H-H Angiotensin II Type 1–Receptor Activating Antibodies in Renal-Allograft Rejection. New Engl J Med (2005) 352:558–69. 10.1056/NEJMoa035717 15703421

[64] Varki A Cummings RD Esko JD Stanley P Hart GW Aebi M Essentials of Glycobiology (2015). Cold Spring Harbor (NY: Cold Spring Harbor Laboratory Press Available at: http://www.ncbi.nlm.nih.gov/books/NBK310274/ (Accessed July 8, 2020). 27010055

[65] Moremen KW Tiemeyer M Nairn AV Vertebrate protein glycosylation: diversity, synthesis and function. Nat Rev Mol Cell Biol (2012) 13:448–62. 10.1038/nrm3383 PMC3934011 22722607

[66] Varki A Biological roles of glycans. Glycobiology (2017) 27:3–49. 10.1093/glycob/cww086 27558841 PMC5884436

[67] Prenc E Pulanic D Pucic-Bakovic M Pezer M Desnica L Vrhovac R Potential of glycosylation research in graft versus host disease after allogeneic hematopoietic stem cell transplantation. Biochim Biophys Acta (BBA) - Gen Subj (2016) 1860:1615–22. 10.1016/j.bbagen.2016.02.015 PMC4907829 26923767

[68] Maverakis E Kim K Shimoda M Gershwin ME Patel F Wilken R Glycans In The Immune system and The Altered Glycan Theory of Autoimmunity: A Critical Review. J Autoimmun (2015) 0:1–13. 10.1016/j.jaut.2014.12.002 PMC4340844 25578468

[69] National Research Council (US) Committee on Assessing the Importance and Impact of Glycomics and Glycosciences Transforming Glycoscience: A Roadmap for the Future (2012). Washington (DC: National Academies Press (US Available at: http://www.ncbi.nlm.nih.gov/books/NBK109958/ (Accessed July 8, 2020). 23270009

[70] Reily C Stewart TJ Renfrow MB Novak J Glycosylation in health and disease. Nat Rev Nephrol (2019) 15:346–66. 10.1038/s41581-019-0129-4 PMC6590709 30858582

[71] Gudelj I Lauc G Pezer M Immunoglobulin G glycosylation in aging and diseases. Cell Immunol (2018) 333:65–79. 10.1016/j.cellimm.2018.07.009 30107893

[72] Šimurina M de Haan N Vučković F Kennedy NA Štambuk J Falck D Glycosylation of Immunoglobulin G Associates With Clinical Features of Inflammatory Bowel Diseases. Gastroenterology (2018) 154:1320–33.e10. 10.1053/j.gastro.2018.01.002 29309774 PMC5880750

[73] van de Geijn FE Wuhrer M Selman MH Willemsen SP de Man YA Deelder AM Immunoglobulin G galactosylation and sialylation are associated with pregnancy-induced improvement of rheumatoid arthritis and the postpartum flare: results from a large prospective cohort study. Arthritis Res Ther (2009) 11:R193. 10.1186/ar2892 20015375 PMC3003510

[74] Lundström SL Hensvold AH Rutishauser D Klareskog L Ytterberg AJ Zubarev RA IgG Fc galactosylation predicts response to methotrexate in early rheumatoid arthritis. Arthritis Res Ther (2017) 19:182. 10.1186/s13075-017-1389-7 28793911 PMC5549282

[75] Gudelj I Salo PP Trbojević-Akmačić I Albers M Primorac D Perola M Low galactosylation of IgG associates with higher risk for future diagnosis of rheumatoid arthritis during 10 years of follow-up. Biochim Biophys Acta Mol Basis Dis (2018) 1864:2034–9. 10.1016/j.bbadis.2018.03.018 29572115

[76] Arnold JN Wormald MR Sim RB Rudd PM Dwek RA The impact of glycosylation on the biological function and structure of human immunoglobulins. Annu Rev Immunol (2007) 25:21–50. 10.1146/annurev.immunol.25.022106.141702 17029568

[77] Dekkers G Treffers L Plomp R Bentlage AEH de Boer M Koeleman CAM Decoding the Human Immunoglobulin G-Glycan Repertoire Reveals a Spectrum of Fc-Receptor- and Complement-Mediated-Effector Activities. Front Immunol (2017) 8:877. 10.3389/fimmu.2017.00877 28824618 PMC5539844

[78] Mihai S Albert H Ludwig RJ Iwata H Björck L Collin M In vivo enzymatic modulation of IgG antibodies prevents immune complex-dependent skin injury. Exp Dermatol (2017) 26:691–6. 10.1111/exd.13163 27512946

[79] Prenc E N-glycosylation of immunoglobulin G in chronic graft-versus-host disease. PhD thesis (2019). Available at: http://medlib.mef.hr/3450/ (Accessed July 8, 2020).

[80] Serody J GVHD and miR: good things in small packages. Blood (2015) 126:1265–7. 10.1182/blood-2015-07-657114 PMC4566807 26359430

[81] Ardekani AM Naeini MM The Role of MicroRNAs in Human Diseases. Avicenna J Med Biotechnol (2010) 2:161–79. PMC3558168 23407304

[82] Garchow B Kiriakidou M MicroRNA-21 deficiency protects from lupus-like autoimmunity in the chronic graft-versus-host disease model of systemic lupus erythematosus. Clin Immunol (2016) 162:100–6. 10.1016/j.clim.2015.11.010 PMC6527104 26631756

[83] Xie L-N Zhou F Liu X-M Fang Y Yu Z Song N-X Serum microRNA155 is increased in patients with acute graft-versus-host disease. Clin Transplant (2014) 28:314–23. 10.1111/ctr.12314 24494749

[84] Ranganathan P Heaphy CEA Costinean S Stauffer N Na C Hamadani M Regulation of acute graft-versus-host disease by microRNA-155. Blood (2012) 119:4786–97. 10.1182/blood-2011-10-387522 PMC3367879 22408260

[85] Wu Y Schutt S Paz K Zhang M Flynn RP Bastian D MicroRNA-17-92 is required for T-cell and B-cell pathogenicity in chronic graft-versus-host disease in mice. Blood (2018) 131:1974–86. 10.1182/blood-2017-06-789321 PMC5921962 29530952

[86] Zhao X Wang Y Lv M Kong Y Luo H Ye X miR-153-3p, a new bio-target, is involved in the pathogenesis of acute graft-versus-host disease via inhibition of indoleamine- 2,3-dioxygenase. Oncotarget (2016) 7:48321–34. 10.18632/oncotarget.10220 PMC5217020 27340781

[87] Ranganathan P Ngankeu A Zitzer NC Leoncini P Yu X Casadei L Serum miR-29a is upregulated in acute Graft versus Host Disease and activates dendritic cells through TLR binding. J Immunol (2017) 198:2500–12. 10.4049/jimmunol.1601778 PMC5340605 28159900

[88] Stickel N Prinz G Pfeifer D Hasselblatt P Schmitt-Graeff A Follo M MiR-146a regulates the TRAF6/TNF-axis in donor T cells during GvHD. Blood (2014) 124(16):2586–95. 10.1182/blood-2014-04-569046 25205119

[89] Leonhardt F Grundmann S Behe M Bluhm F Dumont RA Braun F Inflammatory neovascularization during graft-versus-host disease is regulated by αv integrin and miR-100. Blood (2013) 121:3307–18. 10.1182/blood-2012-07-442665 23327924

[90] Lee C-W Wohlan K Dallmann I Förster R Ganser A Krueger A miR-181a Expression in Donor T Cells Modulates Graft-versus-Host Disease after Allogeneic Bone Marrow Transplantation. JI (2016) 196:3927–34. 10.4049/jimmunol.1502152 27009493

[91] El Andaloussi S Mäger I Breakefield XO Wood MJA Extracellular vesicles: biology and emerging therapeutic opportunities. Nat Rev Drug Discovery (2013) 12:347–57. 10.1038/nrd3978 23584393

[92] Keerthikumar S Chisanga D Ariyaratne D Al Saffar H Anand S Zhao K ExoCarta: A Web-Based Compendium of Exosomal Cargo. J Mol Biol (2016) 428:688–92. 10.1016/j.jmb.2015.09.019 PMC4783248 26434508

[93] Bobrie A Colombo M Raposo G Théry C Exosome secretion: molecular mechanisms and roles in immune responses. Traffic (2011) 12:1659–68. 10.1111/j.1600-0854.2011.01225.x 21645191

[94] Lia G Brunello L Omedè P Astolfi M Drandi D Giaccone L Extracellular Vesicles as Potential Biomarker for Acute Graft-Versus-Host-Disease. Blood (2016) 128:2239–9. 10.1182/blood.V128.22.2239.2239

[95] Lia G Brunello L Bruno S Carpanetto A Omedè P Festuccia M Extracellular vesicles as potential biomarkers of acute graft-vs-host disease. Leukemia (2018) 32:765–73. 10.1038/leu.2017.277 28852198

[96] Valadi H Ekström K Bossios A Sjöstrand M Lee JJ Lötvall JO Exosome-mediated transfer of mRNAs and microRNAs is a novel mechanism of genetic exchange between cells. Nat Cell Biol (2007) 9:654–9. 10.1038/ncb1596 17486113

[97] Stoorvogel W Functional transfer of microRNA by exosomes. Blood (2012) 119:646–8. 10.1182/blood-2011-11-389478 22262739

[98] Crossland RE Norden J Bibby LA Davis J Dickinson AM Evaluation of optimal extracellular vesicle small RNA isolation and qRT-PCR normalisation for serum and urine. J Immunol Methods (2016) 429:39–49. 10.1016/j.jim.2015.12.011 26723490

[99] Crossland RE Norden J Kralj Juric M Pearce KF Lendrem C Bibby LA Serum and Extracellular Vesicle MicroRNAs miR-423, miR-199, and miR-93* As Biomarkers for Acute Graft-versus-Host Disease. Front Immunol (2017) 8:1446. 10.3389/fimmu.2017.01446 29176973 PMC5686047

[100] Yoshizawa S Umezu T Saitoh Y Gotoh M Akahane D Kobayashi C Exosomal miRNA Signatures for Late-Onset Acute Graft-Versus-Host Disease in Allogenic Hematopoietic Stem Cell Transplantation. Int J Mol Sci (2018) 19(2493):1–14. 10.3390/ijms19092493 PMC6164670 30142940

[101] Filipovich AH Weisdorf D Pavletic S Socie G Wingard JR Lee SJ National Institutes of Health Consensus Development Project on Criteria for Clinical Trials in Chronic Graft-versus-Host Disease: I. Diagnosis and Staging Working Group Report. Biol Blood Marrow Transplant (2005) 11:945–56. 10.1016/j.bbmt.2005.09.004 16338616

[102] Xiao B Wang Y Li W Baker M Guo J Corbet K Plasma microRNA signature as a noninvasive biomarker for acute graft-versus-host disease. Blood (2013) 122:3365–75. 10.1182/blood-2013-06-510586 PMC3821726 24041574

[103] Sun Z Hu W Xu J Kaufmann AM Albers AE MicroRNA-34a regulates epithelial-mesenchymal transition and cancer stem cell phenotype of head and neck squamous cell carcinoma in vitro. Int J Oncol (2015) 47:1339–50. 10.3892/ijo.2015.3142 26323460

[104] Bogunia-Kubik K Łacina P Crossland RE Wielińska J Czyż A Ussowicz M Differential expression of miRNAs in chronic Graft-versus-Host Disease. HLA (2020) 95:293–4. 10.1111/tan.13844 36398373

[105] Biedermann BC Vascular endothelium and graft-versus-host disease. Best Pract Res Clin Haematol (2008) 21:129–38. 10.1016/j.beha.2008.02.003 18503981

[106] Almici C Skert C Verardi R Di Palma A Bianchetti A Neva A Changes in circulating endothelial cells count could become a valuable tool in the diagnostic definition of acute graft-versus-host disease. Transplantation (2014) 98:706–12. 10.1097/TP.0000000000000385 25119132

[107] Almici C Skert C Bruno B Bianchetti A Verardi R Di Palma A Circulating endothelial cell count: a reliable marker of endothelial damage in patients undergoing hematopoietic stem cell transplantation. Bone Marrow Transplant (2017) 52:1637–42. 10.1038/bmt.2017.194 28892085

[108] Rank A Nieuwland R Toth B Pihusch V Delker R Hiller E Microparticles for diagnosis of graft-versus-host disease after allogeneic stem transplantation. Transplantation (2011) 92:244–50. 10.1097/TP.0b013e318221d3e9 21629178

[109] Zhang R Wang X Hong M Luo T Zhao M Shen H Endothelial microparticles delivering microRNA-155 into T lymphocytes are involved in the initiation of acute graft-versus-host disease following allogeneic hematopoietic stem cell transplantation. Oncotarget (2017) 8:23360–75. 10.18632/oncotarget.15579 PMC5410310 28423578

[110] Nie D-M Wu Q-L Zheng P Chen P Zhang R Li B-B Endothelial microparticles carrying hedgehog-interacting protein induce continuous endothelial damage in the pathogenesis of acute graft-versus-host disease. Am J Physiol Cell Physiol (2016) 310:C821–835. 10.1152/ajpcell.00372.2015 27009877

[111] Chuang PT McMahon AP Vertebrate Hedgehog signalling modulated by induction of a Hedgehog-binding protein. Nature (1999) 397:617–21. 10.1038/17611 10050855

[112] Kanda S Mochizuki Y Suematsu T Miyata Y Nomata K Kanetake H Sonic hedgehog induces capillary morphogenesis by endothelial cells through phosphoinositide 3-kinase. J Biol Chem (2003) 278:8244–9. 10.1074/jbc.M210635200 12514186

[113] Martino M Fedele R Moscato T Ronco F Optimizing outcomes following allogeneic hematopoietic progenitor cell transplantation in AML: the role of hypomethylating agents. Curr Cancer Drug Targets (2013) 13:661–9. 10.2174/15680096113139990005 23713435

[114] Stamou P Marioli D Patmanidi AL Sgourou A Vittoraki A Theofani EP Simple in vitro generation of human leukocyte antigen-G-expressing T-regulatory cells through pharmacological hypomethylation for adoptive cellular immunotherapy against graft-versus-host disease. Cytotherapy (2017) 19:521–30. 10.1016/j.jcyt.2017.01.004 28162915

[115] Kim S-Y Cho S-G Cho B-S Kim M-S Eom K-S Kim Y-J Azacytidine treatment after discontinuation of immunosuppressants in patients with myelodysplastic syndrome and relapse after allo-SCT at a single center. Bone Marrow Transplant (2010) 45:1375–6. 10.1038/bmt.2009.355 20023706

[116] Czibere A Bruns I Kröger N Platzbecker U Lind J Zohren F 5-Azacytidine for the treatment of patients with acute myeloid leukemia or myelodysplastic syndrome who relapse after allo-SCT: a retrospective analysis. Bone Marrow Transplant (2010) 45:872–6. 10.1038/bmt.2009.266 19820729

[117] Lübbert M Bertz H Wäsch R Marks R Rüter B Claus R Efficacy of a 3-day, low-dose treatment with 5-azacytidine followed by donor lymphocyte infusions in older patients with acute myeloid leukemia or chronic myelomonocytic leukemia relapsed after allografting. Bone Marrow Transplant (2010) 45:627–32. 10.1038/bmt.2009.222 19718057

[118] Holler E Butzhammer P Schmid K Hundsrucker C Koestler J Peter K Metagenomic analysis of the stool microbiome in patients receiving allogeneic stem cell transplantation: loss of diversity is associated with use of systemic antibiotics and more pronounced in gastrointestinal graft-versus-host disease. Biol Blood Marrow Transplant (2014) 20:640–5. 10.1016/j.bbmt.2014.01.030 PMC4973578 24492144

[119] Jenq RR Taur Y Devlin SM Ponce DM Goldberg JD Ahr KF Intestinal Blautia Is Associated with Reduced Death from Graft-versus-Host Disease. Biol Blood Marrow Transplant (2015) 21:1373–83. 10.1016/j.bbmt.2015.04.016 PMC4516127 25977230

[120] Taur Y Jenq RR Perales M-A Littmann ER Morjaria S Ling L The effects of intestinal tract bacterial diversity on mortality following allogeneic hematopoietic stem cell transplantation. Blood (2014) 124:1174–82. 10.1182/blood-2014-02-554725 PMC4133489 24939656

[121] Shimizu E Ogawa Y Saijo Y Yamane M Uchino M Kamoi M Commensal microflora in human conjunctiva; characteristics of microflora in the patients with chronic ocular graft-versus-host disease. Ocul Surf (2019) 17:265–71. 10.1016/j.jtos.2019.02.001 30742990

[122] de Paiva CS Jones DB Stern ME Bian F Moore QL Corbiere S Altered Mucosal Microbiome Diversity and Disease Severity in Sjögren Syndrome. Sci Rep (2016) 6:23561. 10.1038/srep23561 27087247 PMC4834578

[123] Khan MF Wang H Environmental Exposures and Autoimmune Diseases: Contribution of Gut Microbiome. Front Immunol (2019) 10:3094. 10.3389/fimmu.2019.03094 31998327 PMC6970196

[124] Opazo MC Ortega-Rocha EM Coronado-Arrázola I Bonifaz LC Boudin H Neunlist M Intestinal Microbiota Influences Non-intestinal Related Autoimmune Diseases. Front Microbiol (2018) 9:432. 10.3389/fmicb.2018.00432 29593681 PMC5857604

[125] Czaja AJ Factoring the intestinal microbiome into the pathogenesis of autoimmune hepatitis. World J Gastroenterol (2016) 22:9257–78. 10.3748/wjg.v22.i42.9257 PMC5107691 27895415

[126] Dickson RP Erb-Downward JR Huffnagle GB The role of the bacterial microbiome in lung disease. Expert Rev Respir Med (2013) 7:245–57. 10.1586/ers.13.24 PMC4007100 23734647

[127] Cribbs SK Beck JM Microbiome in the pathogenesis of cystic fibrosis and lung transplant-related disease. Transl Res (2017) 179:84–96. 10.1016/j.trsl.2016.07.022 27559681

[128] Willner DL Hugenholtz P Yerkovich ST Tan ME Daly JN Lachner N Reestablishment of recipient-associated microbiota in the lung allograft is linked to reduced risk of bronchiolitis obliterans syndrome. Am J Respir Crit Care Med (2013) 187:640–7. 10.1164/rccm.201209-1680OC 23328523

[129] Hildebrandt GC Granell M Urbano-Ispizua A Wolff D Hertenstein B Greinix HT Recipient NOD2/CARD15 variants: a novel independent risk factor for the development of bronchiolitis obliterans after allogeneic stem cell transplantation. Biol Blood Marrow Transplant (2008) 14:67–74. 10.1016/j.bbmt.2007.09.009 18158963

[130] Touihri M Torjeman L Kaabi H Chabaane M Othman TB Hmida S Bronchiolitis obliterans after allogeneic hematopoietic stem cell transplantation: The effect of NOD2/CARD15 mutations in a Tunisian population. Hum Immunol (2019) 80:163–8. 10.1016/j.humimm.2018.12.005 30552907

[131] Boström L Ringdén O Jacobsen N Zwaan F Nilsson B A European multicenter study of chronic graft-versus-host disease. The role of cytomegalovirus serology in recipients and donors–acute graft-versus-host disease, and splenectomy. Transplantation (1990) 49:1100–5. 10.1097/00007890-199006000-00014 2193443

[132] Górski A Jończyk-Matysiak E Międzybrodzki R Weber-Dąbrowska B Borysowski J “Phage Transplantation in Allotransplantation”: Possible Treatment in Graft-Versus-Host Disease? Front Immunol (2018) 9:941. 10.3389/fimmu.2018.00941 29755481 PMC5933259

